# A Paal–Knorr agent for chemoproteomic profiling of targets of isoketals in cells†

**DOI:** 10.1039/d1sc02230j

**Published:** 2021-10-15

**Authors:** Min-Ran Wang, Jing-Yang He, Ji-Xiang He, Ke-Ke Liu, Jing Yang

**Affiliations:** State Key Laboratory of Proteomics, National Center for Protein Sciences – Beijing, Beijing Proteome Research Center, Beijing Institute of Lifeomics 38 Life Sci. Park Road, Changping District Beijing 102206 China yangjing@ncpsb.org.cn

## Abstract

Natural systems produce various γ-dicarbonyl-bearing compounds that can covalently modify lysine in protein targets *via* the classic Paal–Knorr reaction. Among them is a unique class of lipid-derived electrophiles – isoketals that exhibit high chemical reactivity and critical biological functions. However, their target selectivity and profiles in complex proteomes remain unknown. Here we report a Paal–Knorr agent, 4-oxonon-8-ynal (herein termed ONAyne), for surveying the reactivity and selectivity of the γ-dicarbonyl warhead in biological systems. Using an unbiased open-search strategy, we demonstrated the lysine specificity of ONAyne on a proteome-wide scale and characterized six probe-derived modifications, including the initial pyrrole adduct and its oxidative products (*i.e.*, lactam and hydroxylactam adducts), an enlactam adduct from dehydration of hydroxylactam, and two chemotypes formed in the presence of endogenous formaldehyde (*i.e.*, fulvene and aldehyde adducts). Furthermore, combined with quantitative chemoproteomics in a competitive format, ONAyne permitted global, *in situ*, and site-specific profiling of targeted lysine residues of two specific isomers of isoketals, levuglandin (LG) D2 and E2. The functional analyses reveal that LG-derived adduction drives inhibition of malate dehydrogenase MDH2 and exhibits a crosstalk with two epigenetic marks on histone H2B in macrophages. Our approach should be broadly useful for target profiling of bioactive γ-dicarbonyls in diverse biological contexts.

## Abstract 2

Natural systems produce various γ-dicarbonyl-bearing compounds that can covalently modify lysine in protein targets *via* the classic Paal–Knorr reaction.

## Graphical abstract

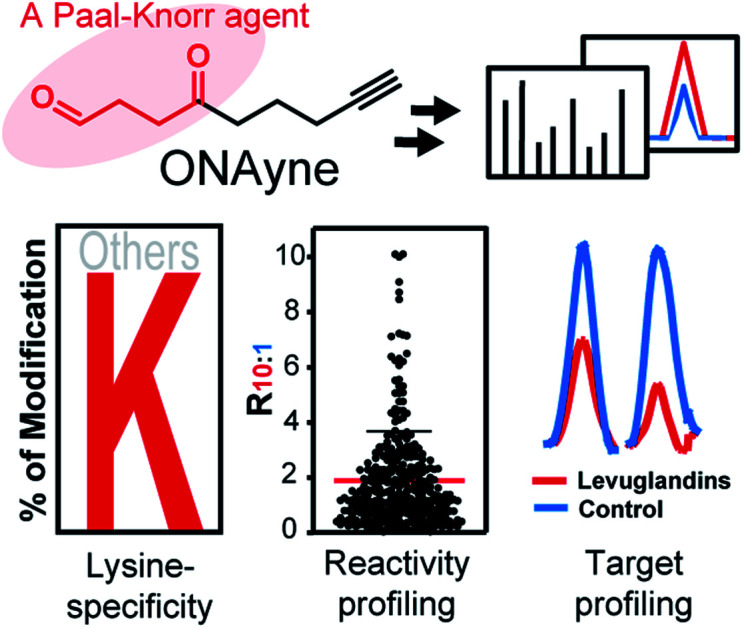

Synthetic chemistry methods have been increasingly underscored by their potential to be repurposed as biocompatible methods for both chemical biology and drug discovery. The most-known examples of such a repurposing approach include the Staudinger ligation^1^ and the Huisgen-based click chemistry.^2^ Moreover, bioconjugation of cysteine and lysine can be built upon facile chemical processes,^3^ while chemoselective labelling of other polar residues (*e.g.*, histidine,^4^ methionine,^5^ tyrosine,^6^ aspartic and glutamic acids^7,8^) requires more elaborate chemistry, thereby offering a powerful means to study the structure and function of proteins, even at a proteome-wide scale.

The classical Paal–Knorr reaction has been reported for a single-step pyrrole synthesis in 1884.^9,10^ The reaction involves the condensation of γ-dicarbonyl with a primary amine under mild conditions (*e.g.*, room temperature, mild acid) to give pyrrole through the intermediary hemiaminals followed by rapid dehydration of highly unstable pyrrolidine adducts (Fig. S1†).

Interestingly, we and others have recently demonstrated that the Paal–Knorr reaction can also readily take place in native biological systems.^11–13^ More importantly, the Paal–Knorr precursor γ-dicarbonyl resides on many endogenous metabolites and bioactive natural products.^14^ Among them of particular interest are isoketals^15^ (IsoKs, also known as γ-ketoaldehydes) which are a unique class of lipid derived electrophiles (LDEs) formed from lipid peroxidation (Fig. S2†)^16^ that has emerged as an important mechanism for cells to regulate redox signalling and inflammatory responses,^17^ and drive ferroptosis,^18^ and this field has exponentially grown over the past few years. It has been well documented that the γ-dicarbonyl group of IsoKs can rapidly and predominantly react with lysine *via* the Paal–Knorr reaction to form a pyrrole adduct *in vitro* (Fig. 1).^15^ Further, the pyrrole formed by IsoKs can be easily oxidized to yield lactam and hydroxylactam products in the presence of molecular oxygen (Fig. 1). These rapid reactions are essentially irreversible. Hence, IsoKs react with protein approximately two orders of magnitude faster than the most-studied LDE 4-hydoxynonenal (4-HNE) that contains α,β-unsaturated carbonyl to generally adduct protein cysteines by Michael addition (Fig. S3†).^15^ Due to this unique adduction chemistry and rapid reactivity, IsoKs exhibit intriguing biological activities, including inhibition of the nucleosome complex formation,^19^ high-density lipoprotein function,^20^ mitochondrial respiration and calcium homeostasis,^21^ as well as activation of hepatic stellate cells.^22^ Furthermore, increases in IsoK-protein adducts have been identified in many major diseases,^23^ such as atherosclerosis, Alzheimer's disease, hypertension and so on.

**Fig. 1:**
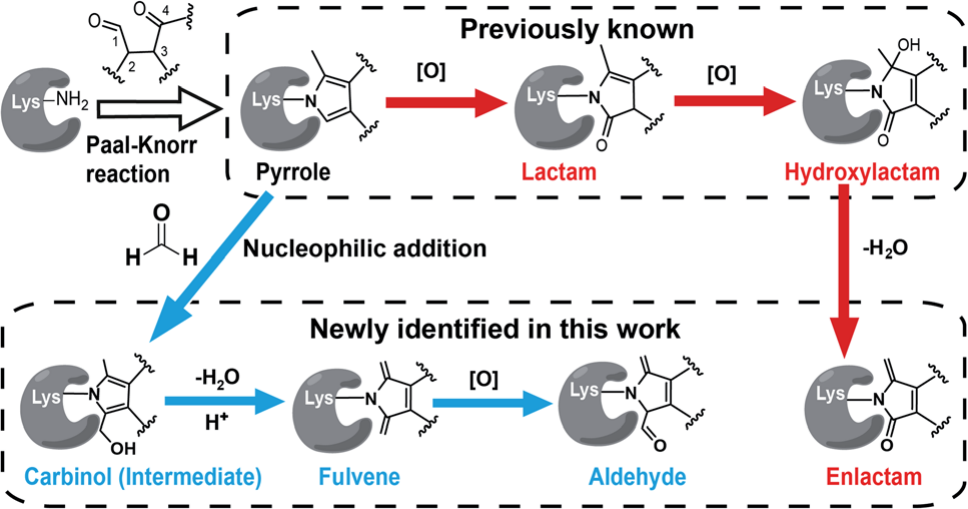
The Paal–Knorr precursor γ-dicarbonyl reacts with the lysine residue on proteins to form diverse chemotypes *via* two pathways. The red arrow shows the oxidation pathway, while the blue one shows the formaldehyde pathway.

Despite the chemical uniqueness, biological significance, and pathophysiological relevance of IsoKs, their residue selectivity and target profiles in complex proteomes remain unknown, hampering the studies of their mechanisms of action (MoAs). Pioneered by the Cravatt group, the competitive ABPP (activity-based protein profiling) has been the method of choice to analyse the molecular interactions between electrophiles (*e.g.*, LDEs,^24^ oncometabolites,^25^ natural products,^26,27^ covalent ligands and drugs^28–30^) and nucleophilic amino acids across complex proteomes. In this regard, many residue-specific chemistry methods and probes have been developed for such studies. For example, several lysine-specific probes based on the activated ester warheads (*e.g.*, sulfotetrafluorophenyl, STP;^31^*N*-hydroxysuccinimide, NHS^32^) have recently been developed to analyse electrophile–lysine interactions at a proteome-wide scale in human tumour cells, which provides rich resources of ligandable sites for covalent probes and potential therapeutics. Although these approaches can also be presumably leveraged to globally and site-specifically profile lysine-specific targets IsoKs, the reaction kinetics and target preference of activated ester-based probes likely differ from those of γ-dicarbonyls, possibly resulting in misinterpretation of ABPP competition results. Ideally, a lysine profiling probe used for a competitive ABPP analysis of IsoKs should therefore possess the same, or at least a similar, warhead moiety. Furthermore, due to the lack of reactive carbonyl groups on IsoK-derived protein adducts, several recently developed carbonyl-directed ligation probes for studying LDE-adductions are also not suitable for target profiling of IsoKs.^33,34^

Towards this end, we sought to design a “clickable” γ-dicarbonyl probe for profiling lysine residues and, in combination with the competitive ABPP strategy, for analysing IsoK adductions in native proteomes. Considering that the diversity of various regio- and stereo- IsoK isomers^15^ (a total of 64, Fig. S2†) in chemical reactivity and bioactivities is likely attributed to the substitution of γ-dicarbonyls at positions 2 and 3, the “clickable” alkyne handle needs to be rationally implemented onto the 4-methyl group in order to minimize the biases when competing with IsoKs in target engagement. Interestingly, we reasoned that 4-oxonon-8-ynal, a previously reported Paal–Knorr agent used as an intermediate for synthesizing fatty acid probes^35^ or oxa-tricyclic compounds,^36^ could be repurposed for the γ-dicarbonyl-directed ABPP application. With this chemical in hand (herein termed ONAyne, Fig. 2A), we first used western blotting to detect its utility in labelling proteins, allowing visualization of a dose-dependent labelling of the proteome *in situ* (Fig. S4†). Next, we set up to incorporate this probe into a well-established chemoproteomic workflow for site-specific lysine profiling *in situ* (Fig. 2A). Specifically, intact cells were labelled with ONAyne *in situ* (200 μM, 2 h, 37 °C, a condition showing little cytotoxicity, Fig. S5†), and the probe-labelled proteome was harvested and processed into tryptic peptides. The resulting probe-labelled peptides were conjugated with both light and heavy azido-UV-cleavable-biotin reagents (1 : 1) *via* Cu^I^-catalyzed azide–alkyne cycloaddition reaction (CuAAC, also known as click chemistry). The biotinylated peptides were enriched with streptavidin beads and photoreleased for LC-MS/MS-based proteomics. The ONAyne-labelled peptides covalently conjugated with light and heavy tags would yield an isotopic signature. We considered only those modified peptide assignments whose MS1 data reflected a light/heavy ratio close to 1.0, thereby increasing the accuracy of these peptide identifications. Using this criterium, we applied a targeted database search to profile three expected probe-derived modifications (PDMs), including 13 pyrrole peptide adducts (Δ273.15), 77 lactam peptide adducts (Δ289.14), and 557 hydroxylactam peptide adducts (Δ305.14), comprising 585 lysine residues on 299 proteins (Fig. S6 and S7†). Among them, the hydroxylactam adducts were present predominately, since the pyrrole formed by this probe, the same as IsoKs, can be easily oxidized when being exposed to O_2_. This finding was in accordance with a previous report where the pyrrole adducts formed by the reaction between IsoK and free lysine could not be detected, but rather their oxidized forms.^37^ Regardless, all three types of adducts were found in one lysine site of EF1A1 (K387, Fig. S8†), further confirming the intrinsic relationship among those adductions *in situ*.

**Fig. 2:**
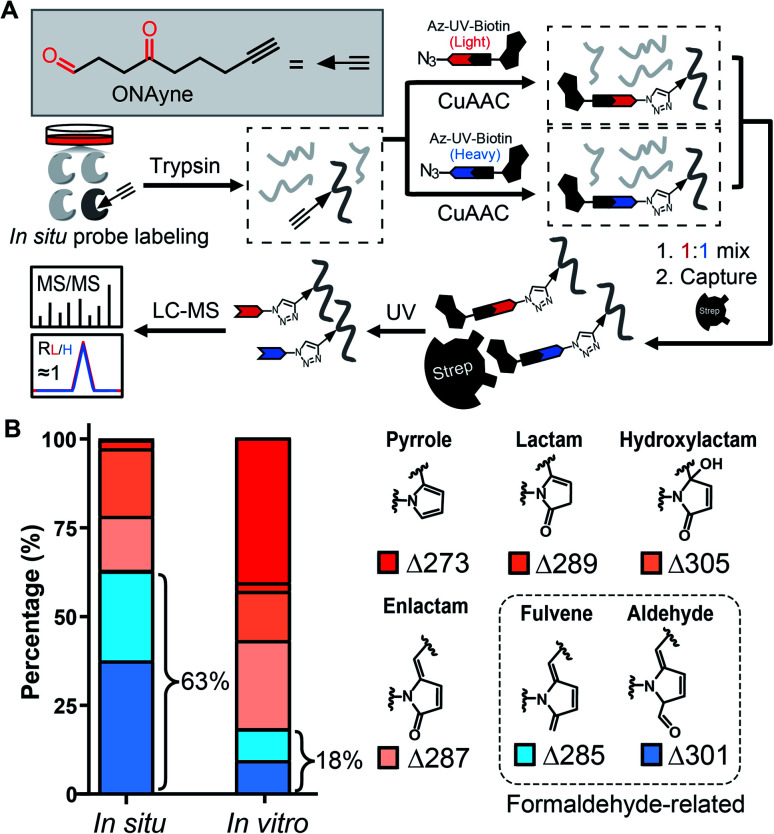
Adduct profile and proteome-wide selectivity of the γ-dicarbonyl probe ONAyne. (A) Chemical structure of ONAyne and schematic workflow for identifying ONAyne-adducted sites across the proteome. (B) Bar chart showing the distribution of six types of ONAyne-derived modifications formed *in situ* and *in vitro* (note: before probe labelling, small molecules in cell lysates were filtered out through desalting columns).

State-of-the-art blind search can offer an opportunity to explore unexpected chemotypes (*i.e.*, modifications) derived from a chemical probe and to unbiasedly assess its proteome-wide residue selectivity.^38,39^ We therefore sought to use one of such tools termed pChem^38^ to re-analyse the MS data (see Methods, ESI†). Surprisingly, the pChem search identified three new and abundant PDMs (Fig. 1 and Table S1†), which dramatically expand the ONAyne-profiled lysinome (2305 sites *versus* 585 sites). Overall, these newly identified PDMs accounted for 74.6% of all identifications (Fig. 2B and Table S2†). Among them, the PDM of Δ287.13 (Fig. 1 and S7†) might be an enlactam product *via* dehydration of the probe-derived hydroxylactam adduct. The other two might be explained by the plausible mechanism as follows (Fig. 1). The endogenous formaldehyde (FA, produced in substantial quantities in biological systems) reacts with the probe-derived pyrrole adduct *via* nucleophilic addition to form a carbinol intermediate, followed by rapid dehydration to a fulvene (Δ285.15, Fig. S7†) and immediate oxidation to an aldehyde (Δ301.14, Fig. S7†). In line with this mechanism, the amount of FA-derived PDMs was largely eliminated when the *in vitro* ONAyne labelling was performed in the FA-less cell lysates (Fig. 2B and Table S3†). Undoubtedly, the detailed mechanisms underlying the formation of these unexpected PDMs require further investigation, and so does the reaction kinetics. Regardless, all main PDMs from ONAyne predominantly target the lysine residue with an average localization probability of 0.77, demonstrating their proteome-wide selectivity (Fig. S9†).

Next, we adapted an ABPP approach to globally and site-specifically quantify the reactivity of lysine towards the γ-dicarbonyl warhead through a dose-dependent labelling strategy (Fig. 3A) that has been proved to be successful for other lysine-specific probes (*e.g.*, STP alkyne).^31^ Specifically, MDA-MB-231 cell lysates were treated with low *versus* high concentrations of ONAyne (1 mM *versus* 0.1 mM) for 1 h. Probe-labelled proteomes were digested into tryptic peptides that were then conjugated to isotopically labelled biotin tags *via* CuAAC for enrichment, identification and quantification. In principle, hyperreactive lysine would saturate labelling at the low probe concentration, whereas less reactive ones would show concentration-dependent increases in labelling. For fair comparison, the STP alkyne-based lysine profiling data were generated by using the same chemoproteomic workflow. Although 77.5% (3207) ONAyne-adducted lysine sites can also be profiled by STP alkyne-based analysis, the former indeed has its distinct target-profile with 930 lysine sites newly identified (Fig. S10 and Table S4†). Interestingly, sequence motif analysis with pLogo^40^ revealed a significant difference in consensus motifs between ONAyne- and STP alkyne-targeting lysines (Fig. S11†).

**Fig. 3:**
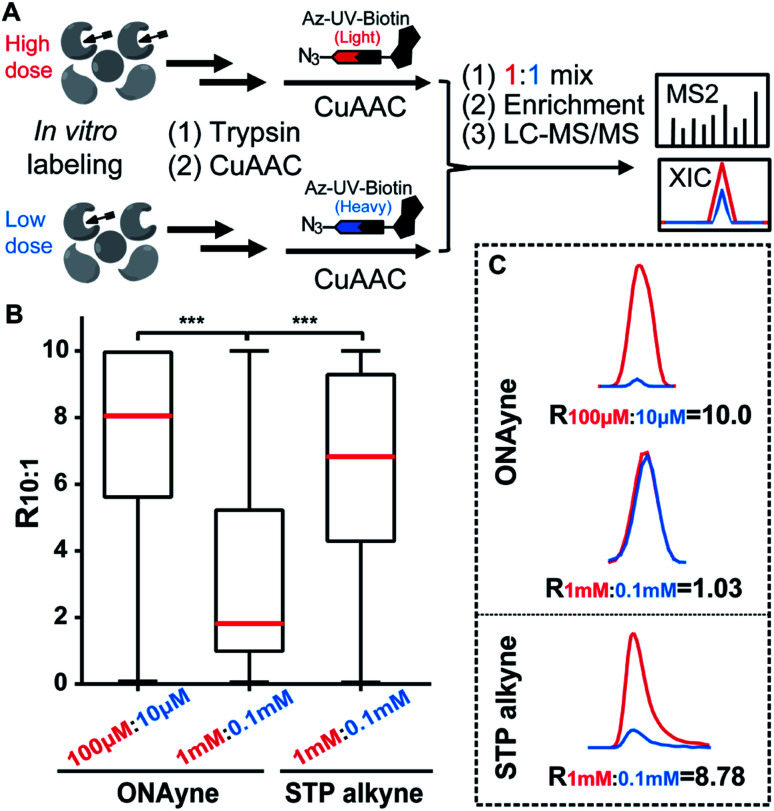
ONAyne-based quantitative reactivity profiling of proteomic lysines. (A) Schematic workflow for quantitative profiling of ONAyne–lysine reactions using the dose-dependent ABPP strategy (B) Box plots showing the distribution of *R*_10:1_ values quantified in ONAyne- and STP alkyne-based ABPP analyses, respectively. Red lines showing the median values. ***p ≤ 0.001 two-tailed Student's *t*-test. (C) Representative extracted ion chromatograms (XICs) showing changes in the EF1A1 peptide bearing K273 that is adducted as indicated, with the profiles for light and heavy-labelled peptides in blue and red, respectively.

Moreover, we quantified the ratio (*R*_1 mM:0.1 mM_) for a total of 2439 ONAyne-tagged lysines (on 922 proteins) and 17904 STP alkyne-tagged lysines (on 4447 proteins) across three biological replicates (Fig. S12 and Table S5†). Strikingly, only 26.7% (651) of quantified sites exhibited nearly dose-dependent increases (*R*_1 mM:0.1 mM_ > 5.0) in reactivity with ONAyne, an indicative of dose saturation (Fig. 3B and C). In contrast, such dose-dependent labelling events accounted for >69.1% of all quantified lysine sites in the STP alkyne-based ABPP analysis.^31^ This finding is in accordance with the extremely fast kinetics of reaction between lysine and γ-dicarbonyls (prone to saturation). Nonetheless, by applying 10-fold lower probe concentrations, overall 1628 (80.2%) detected lysines could be labelled in a fully concentration-dependent manner with the median *R*_10:1_ value of 8.1 (Fig. 3B, C, S12 and Table S5†). Next, we asked whether the dose-depending quantitation data (100 μM *versus* 10 μM) can be harnessed to predict functionality. By retrieving the functional information for all quantified lysines from the UniProt Knowledgebase, we found that those hyper-reactive lysines could not be significantly over-represented with annotation (Fig. S12†). Nonetheless, among all quantified lysines, 509 (25.1%) possess functional annotations, while merely 2.5% of the human lysinome can be annotated. Moreover, 381 (74.8%) ONAyne-labelled sites are known targets of various enzymatic post-translational modifications (PTMs), such as acetylation, succinylation, methylation and so on (Fig. S13†). In contrast, all known PTM sites accounted for only 59.6% of the annotated human lysinome. These findings therefore highlight the intrinsic reactivity of ONAyne towards the ‘hot spots’ of endogenous lysine PTMs.

The aforementioned results validate ONAyne as a fit-for-purpose lysine-specific chemoproteomic probe for competitive isoTOP-ABPP application of γ-dicarbonyl target profiling. Inspired by this, we next applied ONAyne-based chemoproteomics in an *in situ* competitive format (Fig. 4A) to globally profile lysine sites targeted by a mixture of levuglandin (LG) D_2_ and E_2_, two specific isomers of IsoKs that can be synthesized conveniently from prostaglandin H_2_ (ref. 41) (Fig. S2†). Specifically, mouse macrophage RAW264.7 cells (a well-established model cell line to study LDE-induced inflammatory effects) were treated with 2 μM LGs or vehicle (DMSO) for 2 h, followed by ONAyne labelling for an additional 2 h. The probe-labelled proteomes were processed as mentioned above. For each lysine detected in this analysis, we calculated a control/treatment ratio (*R*_C/T_). Adduction of a lysine site by LGs would reduce its accessibility to the ONAyne probe, and thus a higher *R*_C/T_ indicates increased adduction. In total, we quantified 2000 lysine sites on 834 proteins across five biological replicates. Among them, 102 (5.1%) sites exhibited decreases of reactivity towards LGs treatment (*P* < 0.05, Table S6†), thereby being considered as potential targets of LGs. Notably, we found that different lysines on the same proteins showed varying sensitivity towards LGs (*e.g.*, LGs targeted K3 of thioredoxin but not K8, K85 and K94, Table S6†), an indicative of changes in reactivity, though we could not formally exclude the effects of changes in protein expression on the quantified competition ratios. Regardless, to the best of our knowledge, the proteome-wide identification of potential protein targets by IsoKs/LGs has not been possible until this work.

**Fig. 4:**
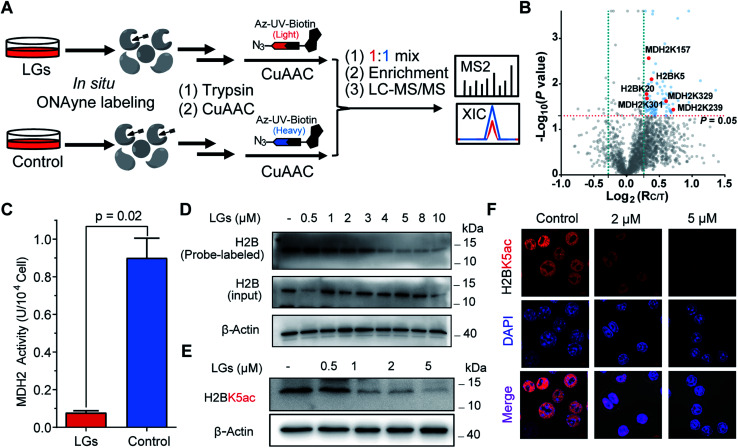
ONAyne-based *in situ* competitive ABPP uncovers functional targets of LGs in macrophages. (A) Schematic workflow for profiling LGs–lysine interactions using ONAyne-based *in situ* competitive ABPP. (B) Volcano plot showing the log_2_ values of the ratio between the control (heavy) and LGs-treated (light) channels and the −log_10_(*P*) of the statistical significance in a two-sample *t*-test for all quantified lysines. Potential targets of LGs are shown in blue (*R*_C/T_>1.2, *P* < 0.05), with the validated ones in red. (C) Bar chart showing the inhibitory effect of 2 μM LGs on the cellular enzymatic activity of MDH2. Data represent means ± standard deviation (*n* = 3). Statistical significance was calculated with two-tailed Student's *t*-tests. (D) Pretreatment of LGs dose-dependently blocked ONAyne-labelling of MDH2 in RAW264.7 cells, as measured by western blotting-based ABPP. (E and F) LGs dose-dependently decreased the H2BK5 acetylation level in RAW 264.7 cells, as measured either by western blotting (E) or by immunofluorescence imaging (F). *n* = 3. For G, nuclei were visualized using DAPI (blue).

We initially evaluated MDH2 (malate dehydrogenase, mitochondrial, also known as MDHM), an important metabolic enzyme that possesses four previously uncharacterized liganded lysine sites (K157, K239, K301 and K329, Fig. 4B) that are far from the active site (Fig. S14†). We found that LGs dramatically reduced the catalytic activity of MDH2 in RAW264.7 cells (Fig. 4C), suggesting a potentially allosteric effect. We next turned our attention to the targeted sites residing on histone proteins, which happen to be modified by functionally important acetylation, including H2BK5ac (Fig. 4B) that can regulate both stemness and epithelial–mesenchymal transition of trophoblast stem cells.^42^ We therefore hypothesized that rapid adduction by LGs competes with the enzymatic formation of this epigenetic mark. Immunoblotting-based competitive ABPP confirmed that LGs dose-dependently blocked probe labelling of H2B (Fig. 4D). Further, both western blots and immunofluorescence assays revealed that LG treatment decreased the level of acetylation of H2BK5 (average *R*_C/T_ = 1.3, *P* = 0.007) in a concentration-dependent manner (Fig. 4E and F). Likewise, a similar competitive crosstalk was observed between acetylation and LG-adduction on H2BK20 (average *R*_C/T_ = 1.2, *P* = 0.01) that is required for chromatin assembly^43^ and/or gene regulation^44^ (Fig. 4B and S15†). Notably, these findings, together with several previous reports by us and others about histone lysine ketoamide adduction by another important LDE, 4-oxo-2-noenal,^11,45,46^ highlight again the potentially important link between lipid peroxidation and epigenetic regulation. In addition to the targets validated as above, many other leads also merit functional studies considering diverse biological or physiologic effects of LGs in macrophages.

## Conclusions

In summary, we have developed a lysine-specific ABPP probe ONAyne that represents a unique addition to the ‘arsenal’ for studying LDEs. Unlike activated ester-based lysine probes,^28,29^ ONAyne offers an interesting lysine-specific chemistry to yield diverse chemotypes *in situ*, particularly regarding the reaction of its pyrrole adduct with endogenous FA. Combined with a competitive ABPP strategy, ONAyne enables us to greatly expand the target spectrum of LGs in RAW264.7 cells. Projecting forward, we envisioned several interesting pursuits with the ONAyne probe that should further address fundamental questions about the MoAs of IsoKs. First, whether and how the regiochemistry and/or stereochemistry of IsoKs lead to distinct electrophile–protein interactions in complex proteomes. To this end, the same chemoproteomic approach described herein (Fig. 4A) offers a convenient target profiling tool for assessing and comparing the competitive lysine-binding of individual IsoK isomers in cells, although here we admit that this effort is not likely to be soon forthcoming, depending on the availability of 64 enantiomerically pure chemicals. Second, whether IsoK-derived lysine adduction is a dynamic process in cells. This question would be presumably addressed by ONAyne-based quantitative chemoproteomics using an established ‘recovery’ setting.^11,47,48^ If yes, discovering an enzymatic mechanism that can afford de-modification will be a task even more technically challenging. Finally, what are the cell-state-specific targets of IsoKs in the more physiologically relevant contexts such as ferroptosis^18^ and inflammatory immune-activation?^49^ The pursuit of the answer to this question may also offer opportunities for basic and translation research purposes. More generally, our approach can also be applied to study many other bioactive γ-dicarbonyls,^14,50–52^ such as dopamine-derived dicatecholaldehyde, natural products (*e.g.*, Ophiobolin A, polygodial, rearranged spongian diterpenes), and reactive metabolites of furan-containing xenobiotics.

## Data availability

The MS data sets have been deposited to the ProteomeXchange Consortium *via* the PRIDE partner repository with the dataset identifiers PXD028270.

## Author contributions

M. R. W. and J. Y. He performed most of experiments and analyzed data. J. X. He performed the pChem search. K. K. L. performed bioinformatic analysis. J. Y. conceived the project, supervised the work, analyzed data and the wrote the manuscript with inputs from others.

## Conflicts of interest

There are no conflicts to declare.

## Supplementary Material

SC-012-D1SC02230J-s001

SC-012-D1SC02230J-s002

SC-012-D1SC02230J-s003

SC-012-D1SC02230J-s004

SC-012-D1SC02230J-s005

SC-012-D1SC02230J-s006

SC-012-D1SC02230J-s007

## References

[cit1] Saxon E., Bertozzi C. R. (2000). Science.

[cit2] Kolb H. C., Finn M. G., Sharpless K. B. (2001). Angew. Chem..

[cit3] Spicer C. D., Davis B. G. (2014). Nat. Commun..

[cit4] Jia S., He D., Chang C. J. (2019). J. Am. Chem. Soc..

[cit5] Lin S., Yang X., Jia S., Weeks A. M., Hornsby M., Lee P. S., Nichiporuk R. V., Iavarone A. T., Wells J. A., Toste F. D., Chang C. J. (2017). Science.

[cit6] Hahm H. S., Toroitich E. K., Borne A. L., Brulet J. W., Libby A. H., Yuan K., Ware T. B., McCloud R. L., Ciancone A. M., Hsu K. L. (2020). Nat. Chem. Biol..

[cit7] Ma N., Hu J., Zhang Z. M., Liu W., Huang M., Fan Y., Yin X., Wang J., Ding K., Ye W., Li Z. (2020). J. Am. Chem. Soc..

[cit8] Bach K., Beerkens B. L. H., Zanon P. R. A., Hacker S. M. (2020). ACS Cent. Sci..

[cit9] Paal C. (1884). Chem. Ber..

[cit10] Knorr L. (1884). Chem Ber.

[cit11] Sun R., Fu L., Liu K., Tian C., Yang Y., Tallman K. A., Porter N. A., Liebler D. C., Yang J. (2017). Mol. Cell. Proteomics.

[cit12] Dasari R., La Clair J. J., Kornienko A. (2017). ChemBioChem.

[cit13] Beuzer P., Axelrod J., Trzoss L., Fenical W., Dasari R., Evidente A., Kornienko A., Cang H., La Clair J. J. (2016). Org. Biomol. Chem..

[cit14] Kornienko A., La Clair J. J. (2017). Nat. Prod. Rep..

[cit15] Davies S. S., Amarnath V., Roberts L. J. (2004). Chem. Phys. Lipids.

[cit16] Yin H., Xu L., Porter N. A. (2011). Chem. Rev..

[cit17] Dias I. H. K., Milic I., Heiss C., Ademowo O. S., Polidori M. C., Devitt A., Griffiths H. R. (2020). Antioxid. Redox Signaling.

[cit18] Jiang X., Stockwell B. R., Conrad M. (2021). Nat. Rev. Mol. Cell Biol..

[cit19] Carrier E. J., Zagol-Ikapitte I., Amarnath V., Boutaud O., Oates J. A. (2014). Biochemistry.

[cit20] May-Zhang L. S., Yermalitsky V., Huang J., Pleasent T., Borja M. S., Oda M. N., Gray Jerome W., Yancey P. G., Linton M. F., Davies S. S. (2018). J. Biol. Chem..

[cit21] Stavrovskaya I. G., Baranov S. V., Guo X., Davies S. S., Roberts L. J., Kristal B. S. (2010). Free Radic. Biol. Med..

[cit22] Longato L., Andreola F., Davies S., Roberts J. L., Fusai G., Pinzani M., Moore K., Rombouts K. (2015). J. Hepatol..

[cit23] Salomon R. G., Bi W. (2015). Antioxid. Redox Signaling.

[cit24] Wang C., Weerapana E., Blewett M. M., Cravatt B. F. (2014). Nat. Methods.

[cit25] Kulkarni R. A., Bak D. W., Wei D., Bergholtz S. E., Chloe A., Shrimp J. H., Alpsoy A., Thorpe A. L., Bavari A. E., Crooks R., Levy M., Florens L., Washburn M. P., Frizzell N., Dykhuizen E. C., Weerapana E., Linehan W. M., Meier J. L. (2019). Nat. Chem. Biol..

[cit26] Spradlin J. N., Hu X., Ward C. C., Brittain S. M., Jones M. D., Ou L., To M., Proudfoot A., Ornelas E., Woldegiorgis M., Olzmann J. A., Bussiere D. E., Thomas J. R., Tallarico J. A., McKenna J. M., Schirle M., Maimone T. J., Nomura D. K. (2019). Nat. Chem. Biol..

[cit27] Tian C., Sun R., Liu K., Fu L., Liu X., Zhou W., Yang Y., Yang J. (2017). Cell Chem. Biol..

[cit28] Backus K. M., Correia B. E., Lum K. M., Forli S., Horning B. D., González-Páez G. E., Chatterjee S., Lanning B. R., Teijaro J. R., Olson A. J., Wolan D. W., Cravatt B. F. (2016). Nature.

[cit29] Vinogradova E. V., Zhang X., Remillard D., Lazar D. C., Suciu R. M., Wang Y., Bianco G., Yamashita Y., Crowley V. M., Schafroth M. A., Yokoyama M., Konrad D. B., Lum K. M., Simon G. M., Kemper E. K., Lazear M. R., Yin S., Blewett M. M., Dix M. M., Nguyen N., Shokhirev M. N., Chin E. N., Lairson L. L., Melillo B., Schreiber S. L., Forli S., Teijaro J. R., Cravatt B. F. (2020). Cell.

[cit30] Senkane K., Vinogradova E. V., Suciu R. M., Crowley V. M., Zaro B. W., Bradshaw J. M., Brameld K. A., Cravatt B. F. (2019). Angew. Chem..

[cit31] Hacker S. M., Backus K. M., Lazear M. R., Forli S., Correia B. E., Cravatt B. F. (2017). Nat. Chem..

[cit32] Ward C. C., Kleinman J. I., Nomura D. K. (2017). ACS Chem. Biol..

[cit33] Chen Y., Liu Y., Lan T., Qin W., Zhu Y., Qin K., Gao J., Wang H., Hou X., Chen N., Friedmann Angeli J. P., Conrad M., Wang C. (2018). J. Am. Chem. Soc..

[cit34] Chen Y., Cong Y., Quan B., Lan T., Chu X., Ye Z., Hou X., Wang C. (2017). Redox Biol.

[cit35] Niphakis M. J., Lum K. M., Cognetta A. B., Correia B. E., Ichu T. A., Olucha J., Brown S. J., Kundu S., Piscitelli F., Rosen H., Cravatt B. F. (2015). Cell.

[cit36] Bao W., Tao Y., Cheng J., Huang J., Cao J., Zhang M., Ye W., Wang B., Li Y., Zhu L., Lee C. S. (2018). Org. Lett..

[cit37] Brame C. J., Salomon R. G., Morrow J. D., Roberts L. J. (1999). J. Biol. Chem..

[cit38] He J. X., Fei Z. C., Fu L., Tian C. P., He F. C., Chi H., Yang J. (2021). BioRxiv.

[cit39] Zanon P. R. A., Yu F., Musacchio P. Z., Lewald L., Zollo M., Krauskopf K., Mrdovic D., Raunft P., Maher T. E., Cigler M., Chang C. J., Lang K., Toste F. D., Nesvizhiskii A. I., Hacker S. M. (2021). ChemRxiv.

[cit40] O'Shea J. P., Chou M. F., Quader S. A., Ryan J. K., Church G. M., Schwartz D. (2013). Nat. Methods.

[cit41] Davies S. S., Amarnath V., Brame C. J., Boutaud O., Roberts L. J. (2007). Nat. Protoc..

[cit42] Abell A. N., Jordan N. V., Huang W., Prat A., Midland A. A., Johnson N. L., Granger D. A., Mieczkowski P. A., Perou C. M., Gomez S. M., Li L., Johnson G. L. (2011). Cell Stem Cell.

[cit43] Ruiz P. D., Gamble M. J. (2018). Nat. Commun..

[cit44] Kumar V., Rayan N. A., Muratani M., Lim S., Elanggovan B., Xin L., Lu T., Makhija H., Poschmann J., Lufkin T., Ng H. H., Prabhakar S. (2016). Genome Res..

[cit45] Galligan J. J., Rose K. L., Beavers W. N., Hill S., Tallman K. A., Tansey W. P., Marnett L. J. (2014). J. Am. Chem. Soc..

[cit46] Jin J., He B., Zhang X., Lin H., Wang Y. (2016). J. Am. Chem. Soc..

[cit47] Akter S., Fu L., Jung Y., Lo Conte M., Lawson J. R., Lowther W. T., Sun R., Liu K., Yang J., Carroll K. S. (2018). Nat. Chem. Biol..

[cit48] Yang J., Tallman K. A., Porter N. A., Liebler D. C. (2015). Anal. Chem..

[cit49] Muri J., Kopf M. (2021). Nat. Rev. Immunol..

[cit50] Cho H., Shen Q., Zhang L. H., Okumura M., Kawakami A., Ambrose J., Sigoillot F., Miller H. R., Gleim S., Cobos-Correa A., Wang Y., Piechon P., Roma G., Eggimann F., Moore C., Aspesi Jr. P., Mapa F. A., Burks H., Ross N. T., Krastel P., Hild M., Maimone T. J., Fisher D. E., Nomura D. K., Tallarico J. A., Canham S. M., Jenkins J. L., Forrester W. C. (2021). Cell Chem. Biol..

[cit51] Lin D., Li C., Peng Y., Gao H., Zheng J. (2014). Drug Metab. Dispos..

[cit52] Druckova A., Mernaugh R. L., Ham A. J. L., Marnett L. J. (2007). Chem. Res. Toxicol..

