# Supplementary material for: A Paal–Knorr agent for chemoproteomic profiling of targets of isoketals in cells
Source: Chem Sci. 2021 Oct 15;12(43):14557–63. doi: 10.1039/d1sc02230j (PMC8580055; doi:10.1039/d1sc02230j)
Supplement: SC-012-D1SC02230J-s001 [file SC-012-D1SC02230J-s001.pdf]

Electronic Supplementary Material (ESI) for Chemical Science.
This journal is © The Royal Society of Chemistry 2021

SUPPLEMENTARY INFORMATION

A Paal-Knorr agent for chemoproteomic profiling of targets of isoketals in cells

Min-Ran Wang[a†], Jing-Yang He[a†], Ji-Xiang He[a], Keke Liu[a], Jing Yang[*a]

[a]State Key Laboratory of Proteomics, National Center for Protein Sciences • Beijing, Beijing Proteome Research Center, Beijing Institute of Lifeomics, 38 Life Sci. Park Road, Changping District, Beijing 102206, China

[*]E-mail: yangjing@ncpsb.org.cn

[†]These authors contributed equally.

Table of Contents

Figure S1 4
Figure S2 5
Figure S3 6
Figure S4 7
Figure S5 8
Figure S6 9
Figure S7 10
Figure S8 11
Figure S9 12
Figure S10 13
Figure S11 14
Figure S12 15
Figure S13 16
Figure S14 17
Figure S15 18
Legends to Supplemental Tables 19
Table S1. Reporting summary by pChem search of the data generated from ONAyne-based chemoproteomics 19
Table S2. Site-specific mapping of ONAyne-derived modifications in intact cells 19
Table S3. Site-specific mapping of ONAyne-derived modifications in cell lysates 19
Table S4. Overlaps of the lysinome mapped by using ONAyne and STP alkyne 19
Table S5. Quantitative reactivity profiling of proteomic lysines 20
Table S6. Proteome-wide mapping LGs-targeting lysines in RAW264.7 cells 20
Methods 21
Reagents and antibodies 21
Cell culture and treatment 21
Cytotoxicity assay 21
ONAyne labeling 22
Preparing the ONAyne-labeled protein samples 22
Liquid chromatography-tandem mass spectrometry (LC-MS/MS) 23
pChem analysis 23

Targeted identification and quantification 24
Motif analysis 24
MDH2 activity assay 24
Immunoprecipitation 24
Western blotting 25
Immunofluorescence imaging 25
Synthesis of ONAyne 26
NMR spectra 28
EI-MS spectrum 29
References 30

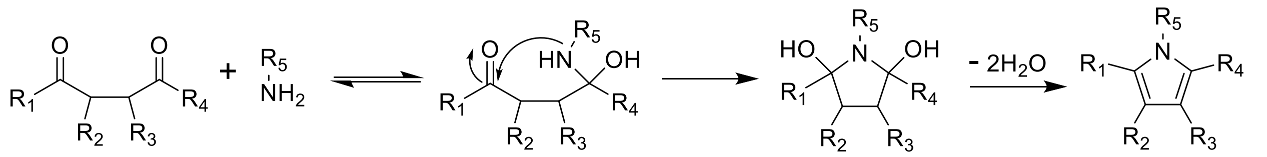

Figure S1. The Paal-Knorr reaction.

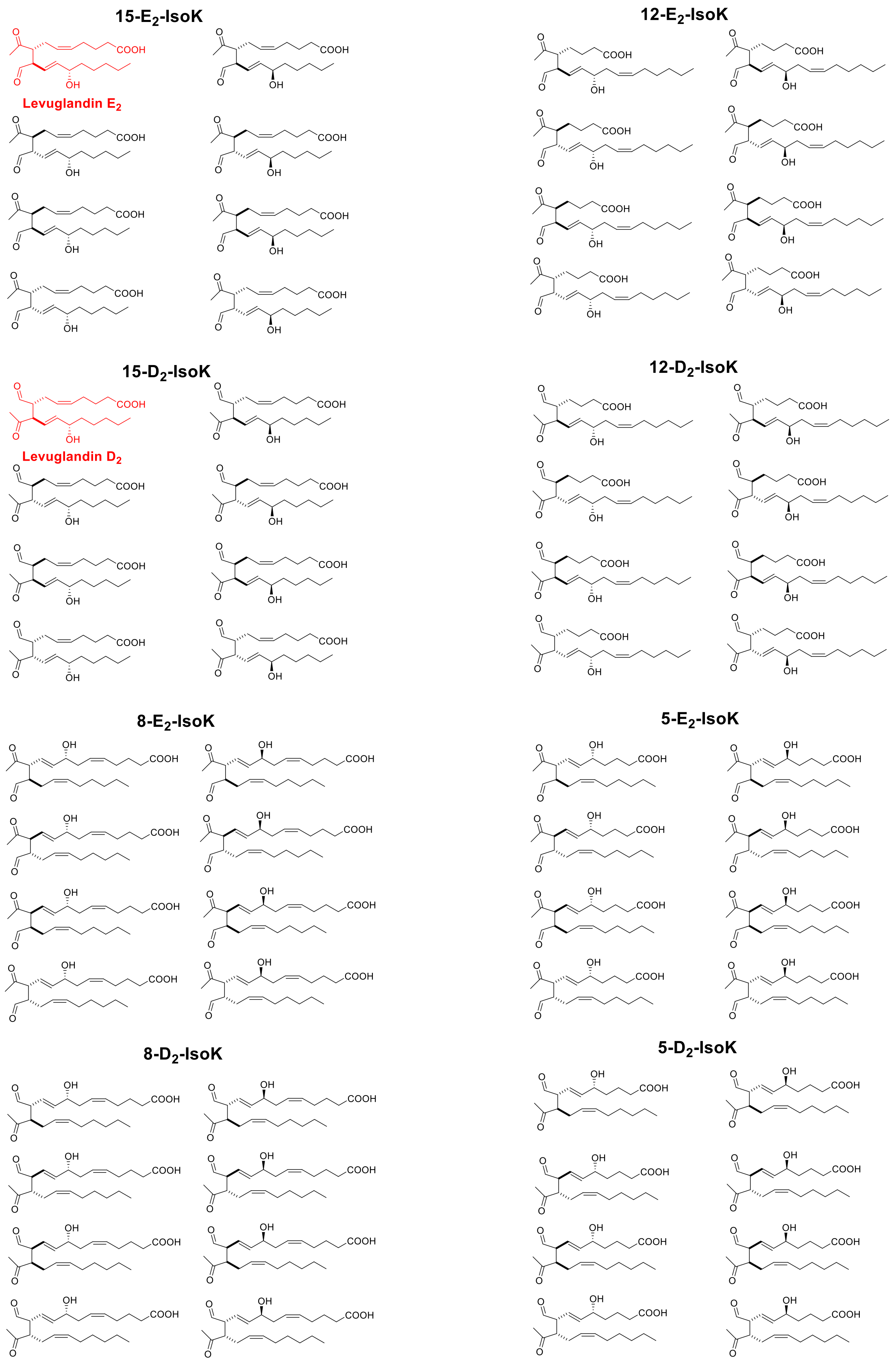

Figure S2. Chemical structures of 64 IsoK isomers.

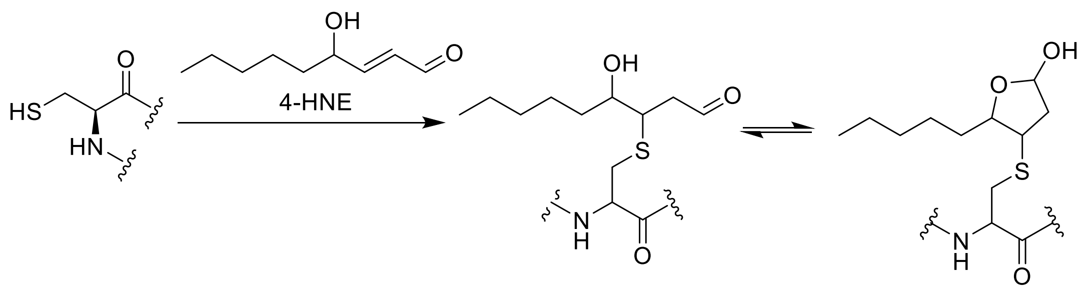

Figure S3. Reaction between 4-HNE and protein cysteine.

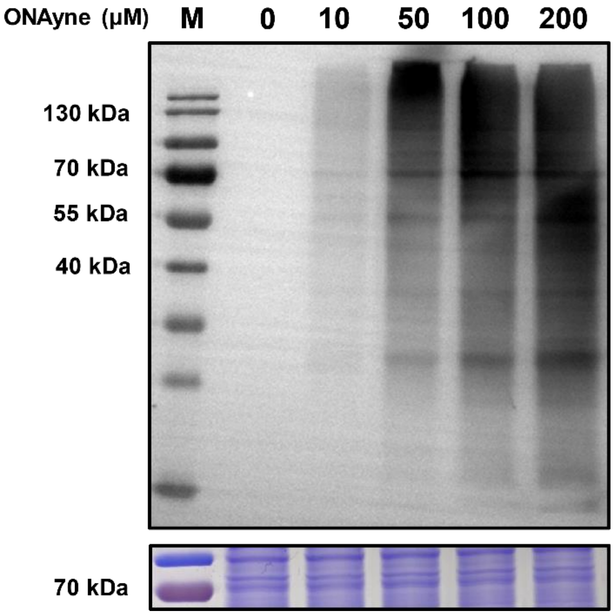

Figure S4. Western blotting detection of ONAyne-derived adduction in the RAW264.7 proteome.

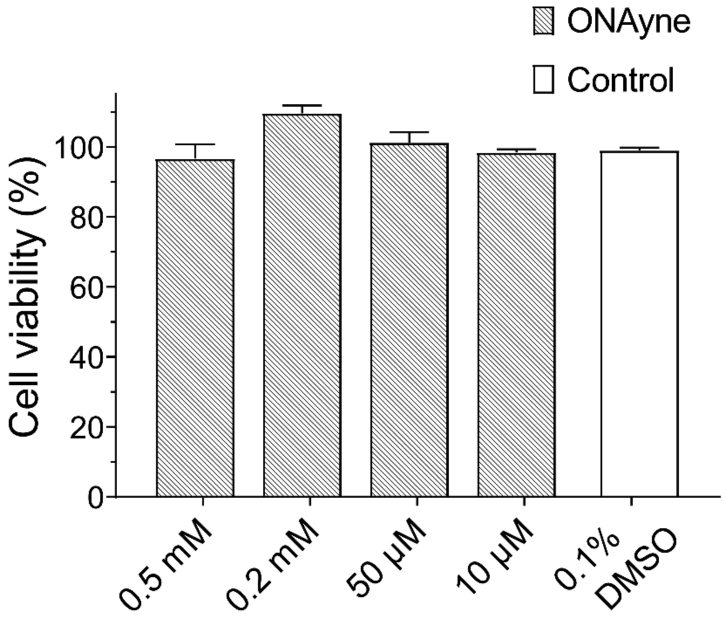

Figure S5. Bar charts showing the effect of ONAyne on cell viability, measured by MTT assay. RAW264.7 cells were treated with vehicle (0.1% DMSO) or ONAyne at increasing concentrations for 2 h. Data represent means ± standard deviation for five independent experiments.

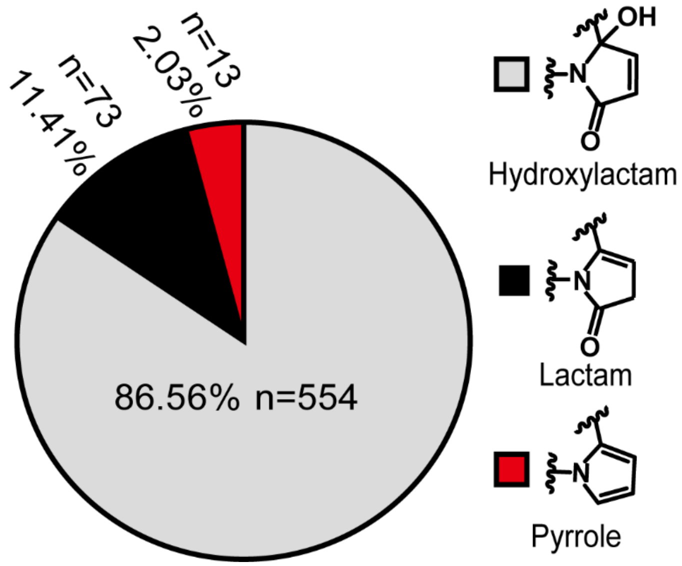

Figure S6. Pie chart showing the distribution of three previously known types of ONAyne-derived peptide adducts (Incl. pyrrole, lactam and hydroxylactam). Data was generated by the pFind-based targeted database search.

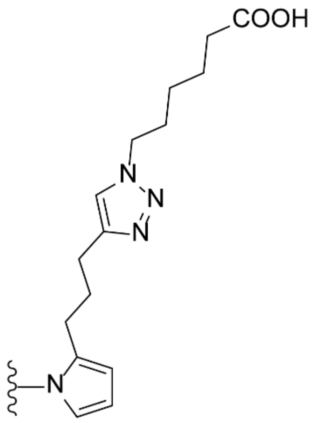

Pyrrole
Chemical Formula: $C_{15}H_{19}N_3O_2$
Exact Mass: 273.14773

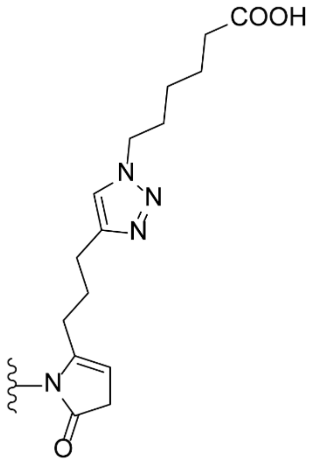

Lactam
Chemical Formula: $C_{15}H_{19}N_3O_3$
Exact Mass: 289.14264

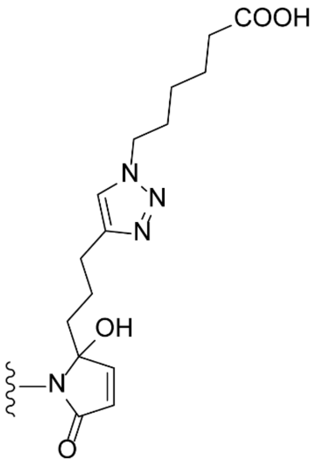

Hydroxylactam
Chemical Formula: $C_{15}H_{19}N_3O_4$
Exact Mass: 305.13756

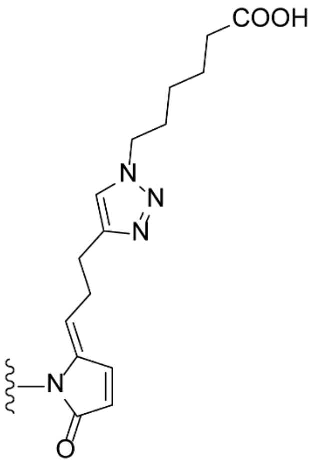

Enlactam
Chemical Formula: $C_{15}H_{17}N_3O_3$
Exact Mass: 287.12699

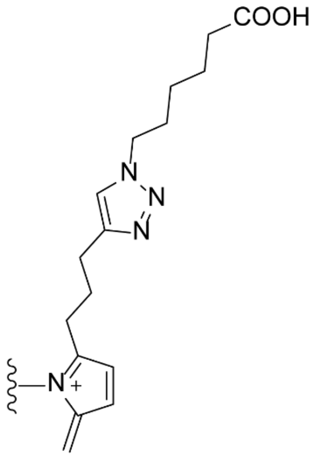

Fulvene
Chemical Formula: $C_{16}H_{19}N_3O_2$
Exact Mass: 285.14773

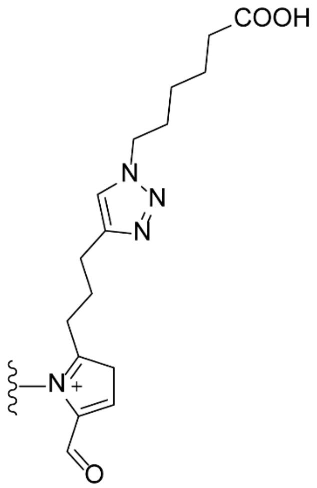

Aldehyde
Chemical Formula: $C_{16}H_{19}N_3O_3$
Exact Mass: 301.14264

Figure S7. Chemical structures of ONAyne-derived lysine modifications.

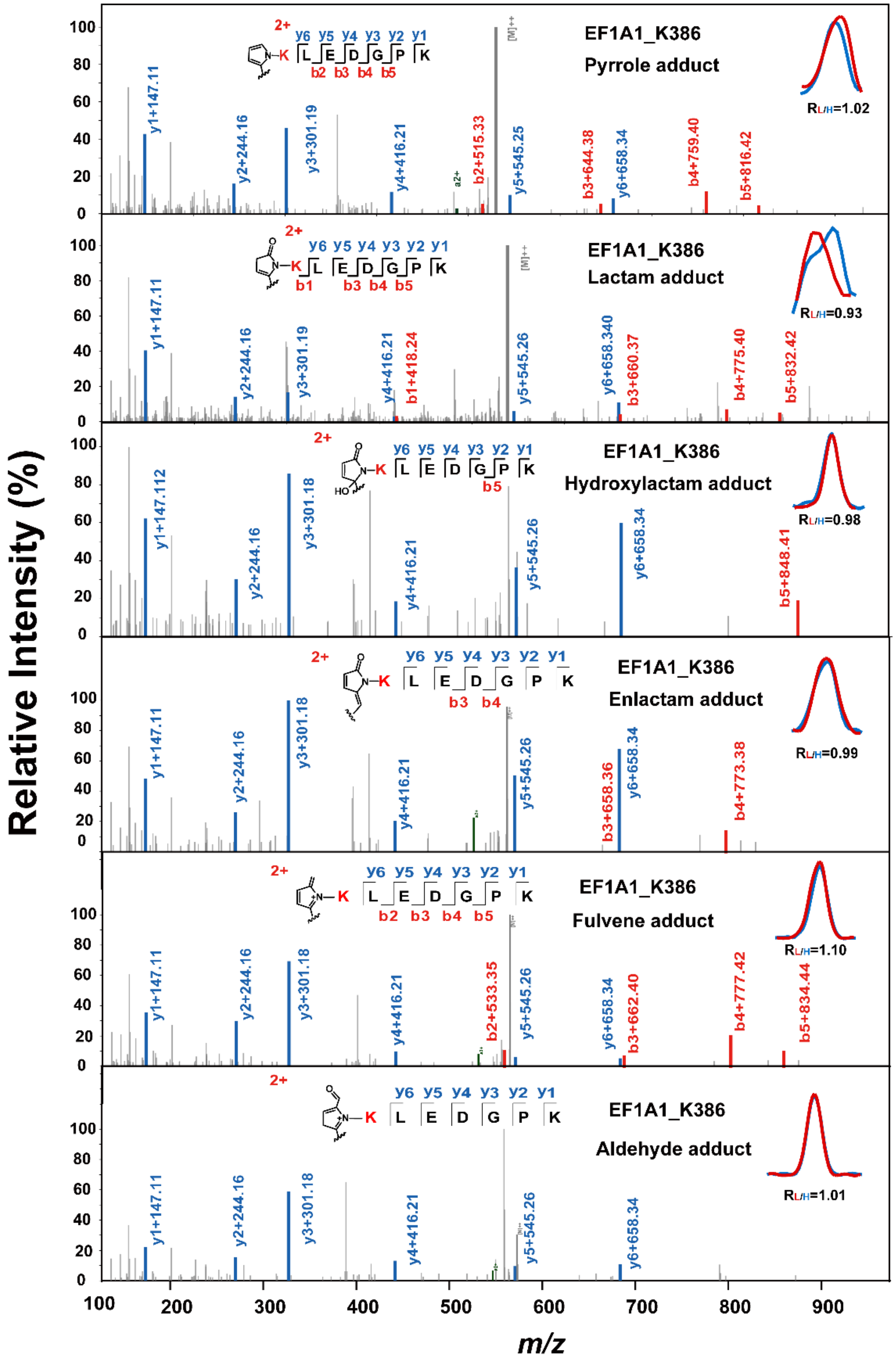

Figure S8. HCD MS/MS spectra of the peptide containing EF1A1^{K387} labeled by six types of ONAyne-derived modifications. Insets: Representative XICs showing for changes in ONAyne-adducted peptides, with the profiles for light and heavy-labeled peptides in blue and red, respectively.

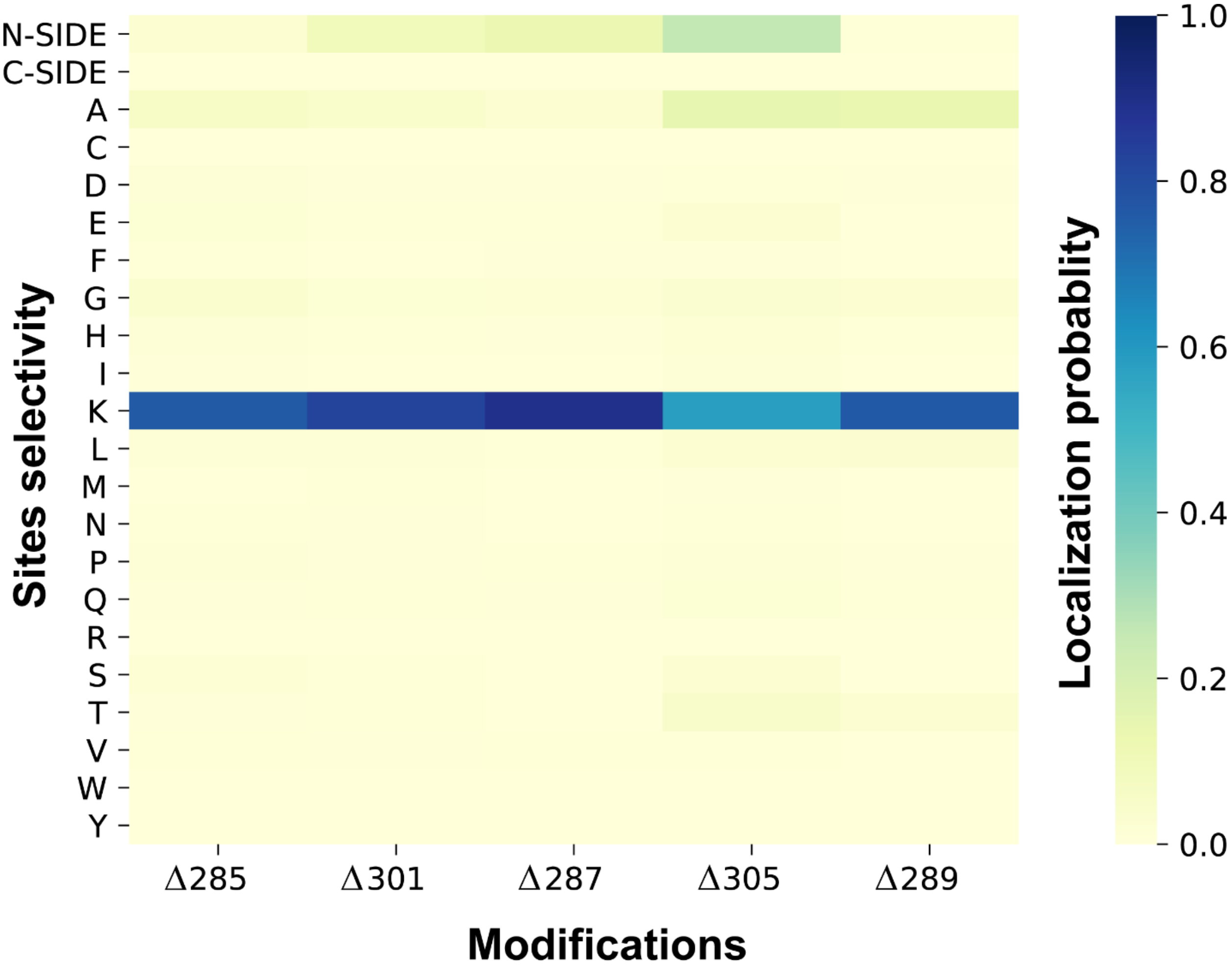

Figure S9. Representative heatmaps generated by the pChem search showing the amino acid localization distribution of the ONAyne-derived modifications. Note: the minor pyrrole adduct was not detected by this open-search.

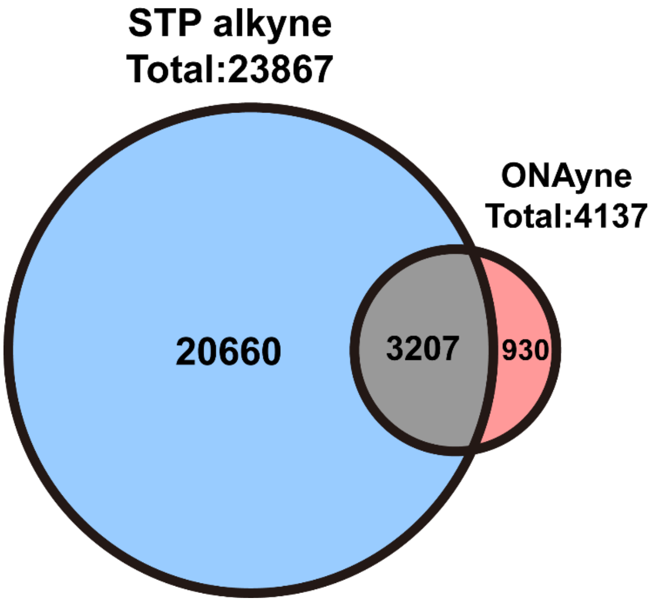

Figure S10. Venn diagram showing the overlap of lysines identified by indicated probes in MDA-MB-231 cell lysates.

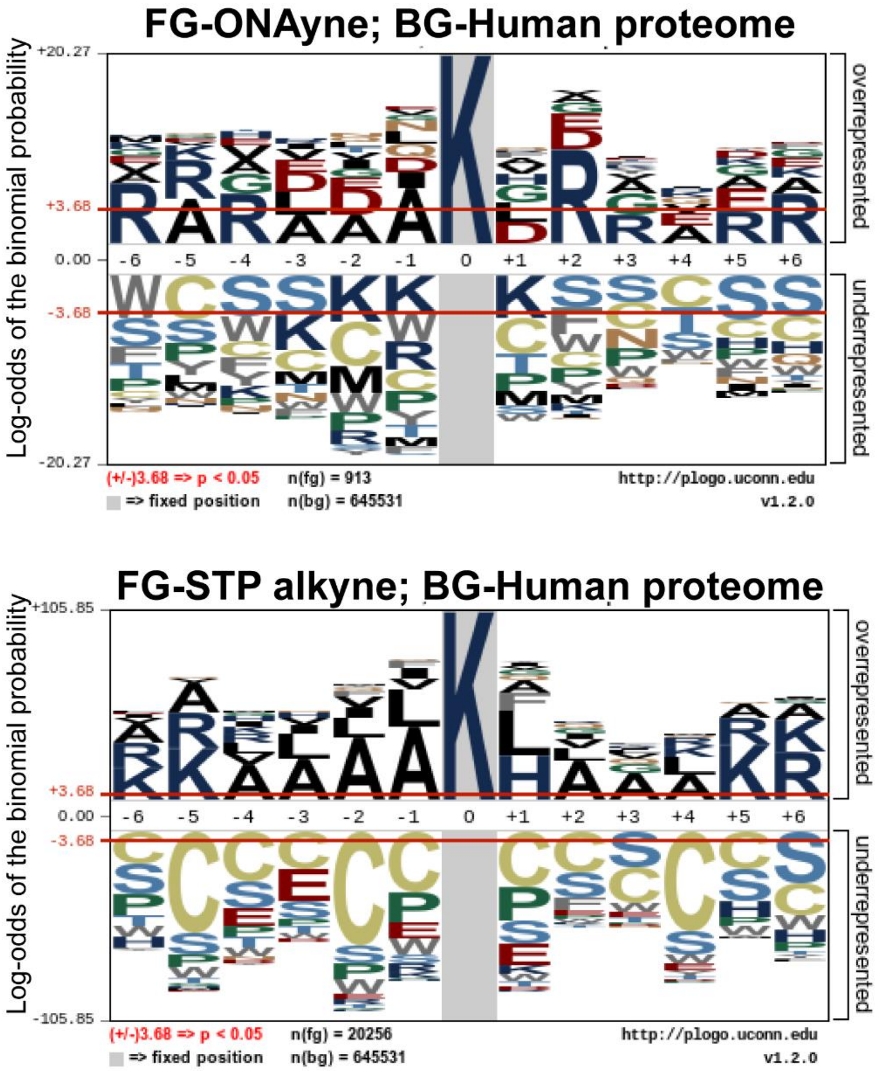

Figure S11. Sequence motif analysis of hyperreactive lysines. Comparison of calculated sequence motif for the lysines specifically labeled by ONAyne (913, human, upper) and those by STP alkyne-based (20256, human, lower). Images were generated with pLogo and scaled to the height of the largest column within the sequence visualization. The red horizontal lines on the pLogo plots denote p=0.05 thresholds.

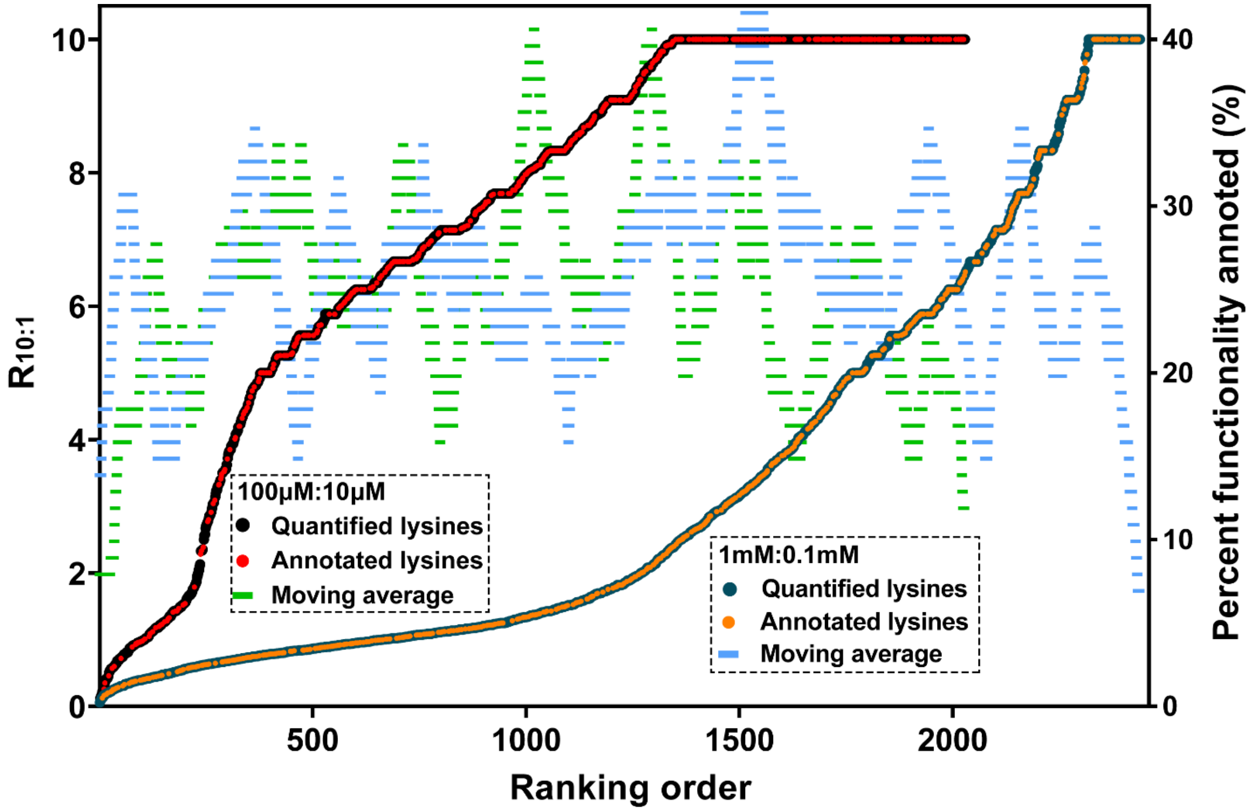

Figure S12. Correlation of $R_{10:1}^{ONAyne}$ values quantified with functional annotations from UniProt, where annotated lysines are shown in red, and all other quantified are in black. Moving average line of functional annotated sites are shown in dashed lines. Quantitation data were generated with ONAyne-based lysine profiling in a concentration-dependent format by comparing 1 mM to 0.1 mM or by comparing 100 μM to 10 μM.

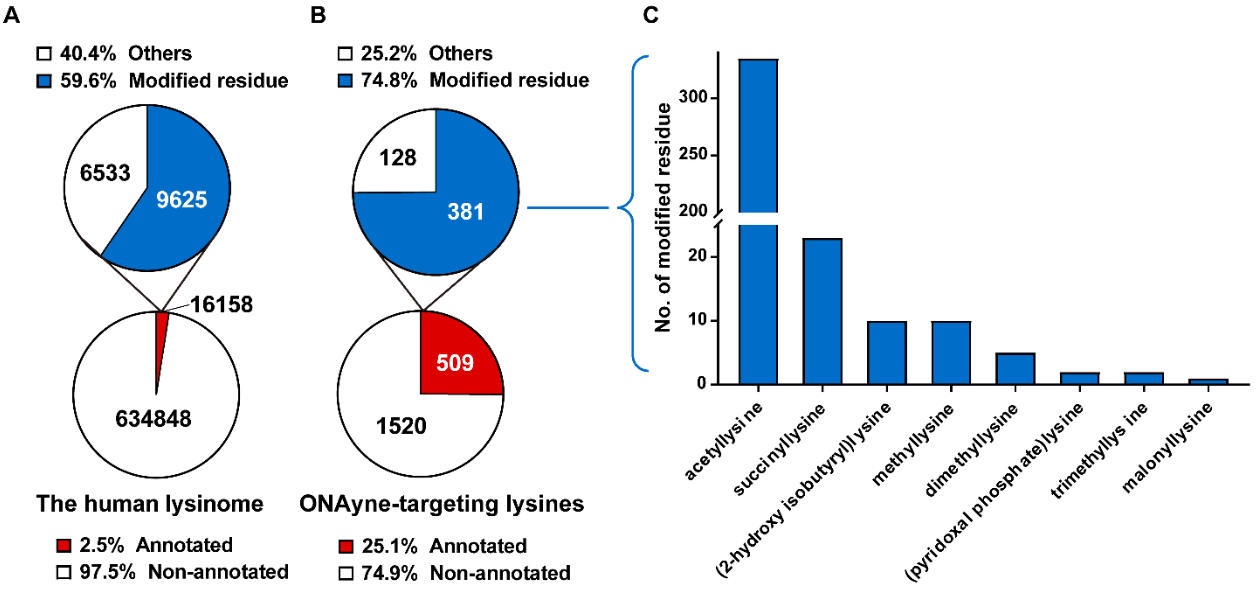

Figure S13. Pie charts showing the classification of functionally annotated (Uniprot) lysines in the human proteome (**A**) and those labeled by ONAyne (**B**). (**C**) Bar chart showing the colocalization of a group of ONAyne adductions with endogenous lysine PTMs. Functional information is retrieved from the Uniprot knowledgebase.

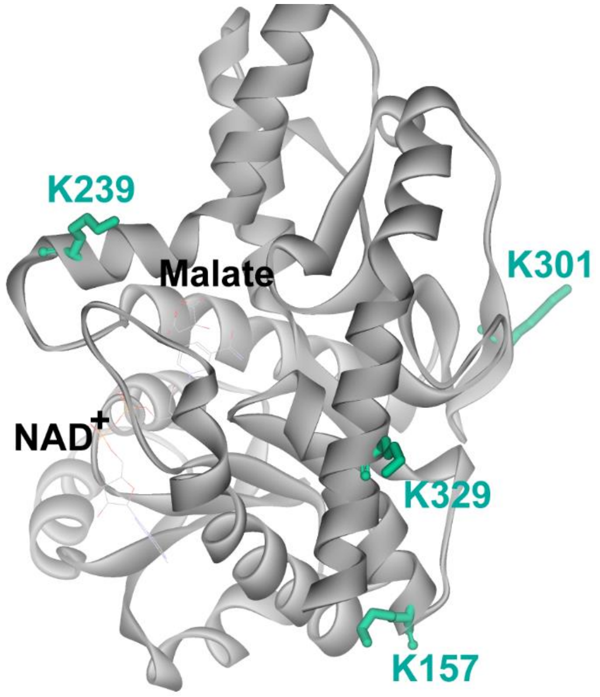

Figure S14. 3D protein structure of MDH2 (PDB#: 2DFD, human), visualized with Discovery Studio 2.5. Targeted lysines are shown in green.

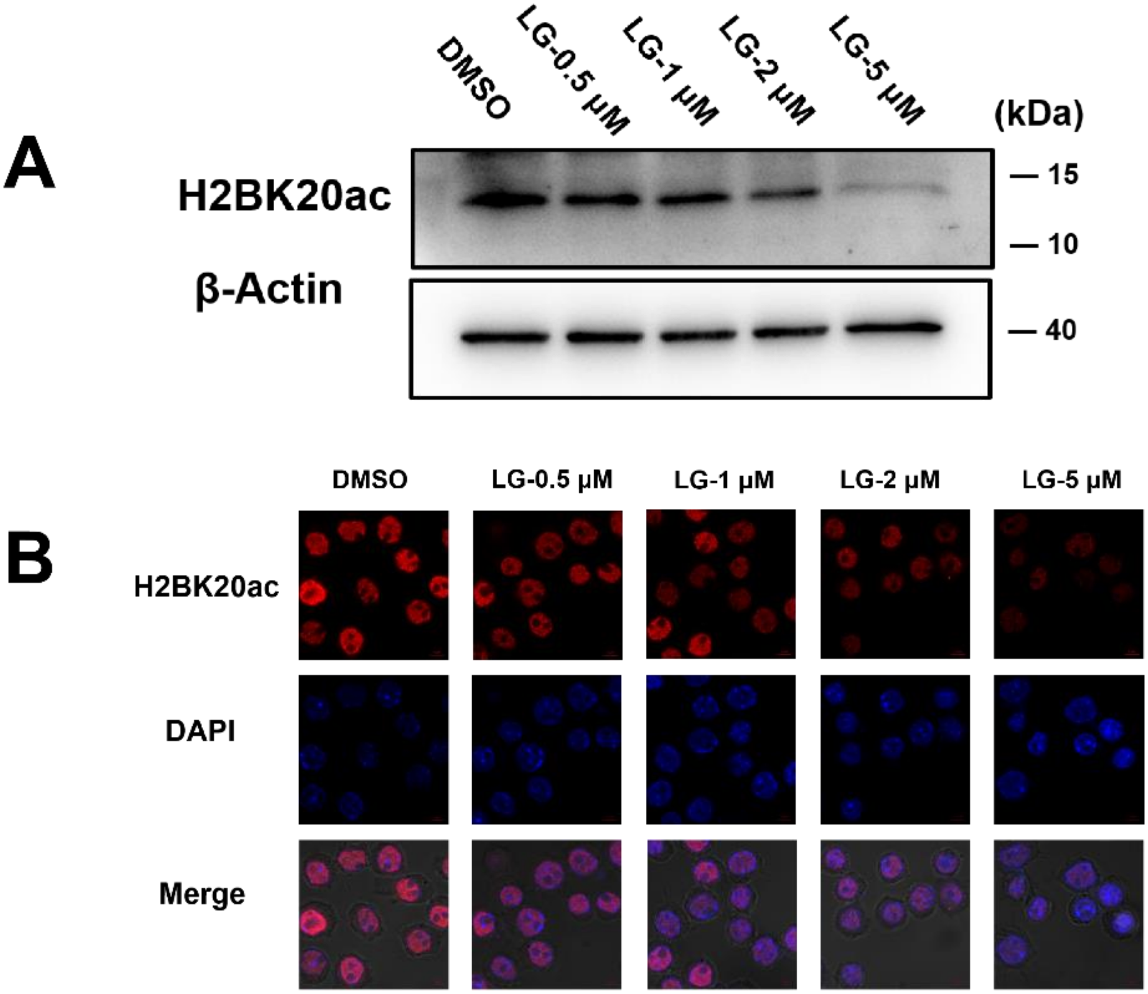

Figure S15. Crosstalk between LGs-derived adduction and lysine acetylation on K20 of H2B. (**A**) Representative Western blot showing the dose-dependent inhibitory effect of LGs-treatment on acetylation at position K20 of H2B (n=3). (**B**) Representative immunofluorescence images showing the effect of LGs-treatment on the levels of H2BK20ac (red, Alexa Fluor 594) in RAW 264.7 cells (n=3). Nuclei were visualized using 4′-6-diamidino-2-phenylindole, dihydrochloride (blue, DAPI).

Legends to Supplemental Tables

Table S1. Reporting summary by pChem search of the data generated from ONAyne-based chemoproteomics. RAW264.7 cells were incubated with 200 µM ONAyne for 2h. The ONAyne-labeled proteome was harvested and then processed into tryptic peptides. The resulting probe-labeled peptides were aliquoted equally and conjugated with light and heavy azido-UV-cleavable-biotin (Az-UV-biotin) reagents (1:1) via CuAAC reaction. The light and heavy 'Click' reaction mixtures were mixed equally in amount, and cleaned with SCX. The biotinylated peptides were captured with streptavidin and photoreleased for LC-MS/MS analysis. Data was search with pChem and exported in the reporting summary shown here as it is.

Table S2. Site-specific mapping of ONAyne-derived modifications in intact cells. Data generated for **Table S1** were searched with pFind 3.0 against known probe-derived modifications and those newly identified by pChem (**Fig. S7**). The ONAyne-labeled peptides covalently conjugated with light and heavy tags would yield an isotopic signature. Only those modified peptide assignments whose MS1 data reflected a light/heavy ratio close to 1.0 (less than 1.5-fold change) were considered as true hits. Quantitation was performed by pQuant and the results were obtained from three biological replicates with two LC-MS/MS runs for each.

Table S3. Site-specific mapping of ONAyne-derived modifications in cell lysates. RAW264.7 cell lysates were desalted to filter out endogenous formaldehyde and then incubated with 200 µM ONAyne at room temperature (~25°C) for 1h. The ONAyne-labeled proteome was then processed into tryptic peptides. The resulting probe-labeled peptides were aliquoted equally and conjugated with light and heavy azido-UV-cleavable-biotin (Az-UV-biotin) reagents (1:1) via CuAAC reaction. The light and heavy 'Click' reaction mixtures were mixed equally in amount, and cleaned with SCX. The biotinylated peptides were captured with streptavidin and photoreleased for LC-MS/MS analysis. Data were searched with pFind 3.0 against known probe-derived modifications and those newly identified by pChem (**Fig. S7**). The ONAyne-labeled peptides covalently conjugated with light and heavy tags would yield an isotopic signature. Only those modified peptide assignments whose MS1 data reflected a light/heavy ratio close to 1.0 (less than 1.5-fold change) were considered as true hits. Quantitation was performed by pQuant and the results were obtained from one biological replicate with two LC-MS/MS runs.

Table S4. Overlaps of the lysinome mapped by using ONAyne and STP alkyne. MDA-MB-231 cell lysates were labeled with ONAyne or STP alkyne, respectively, using two different doses

(0.1 and 1 mM), and processed into tryptic peptides. The ONAyne-modified peptides were further conjugated to light and heavy azido biotin reagents with a photocleavable linker (Az-UV-biotin), respectively, via CuAAC reaction. The heavy (0.1 mM) and light (1 mM) labeled samples then were mixed equally in amount, cleaned with SCX and subjected to streptavidin-based enrichment. After several washing steps, the modified peptides were selectively eluted from beads under 365 nm wavelength of UV light and subjected to LC-MS/MS-based proteomic analysis.

Table S5. Quantitative reactivity profiling of proteomic lysines. MDA-MB-231 cell lysates were labeled with the indicated probes (ONAyne or STP) and labeling concentrations (For ONAyne, 1 mM vs 0.1 mM, 100 µM vs 10 µM; For STP, 1 mM vs 0.1 mM), respectively, and processed into tryptic peptides. The probe-modified peptides were further conjugated to light and heavy azido biotin reagents with a photocleavable linker (Az-UV-biotin), respectively, via CuAAC reaction. The light and heavy labeled samples then were mixed equally in amount, cleaned with SCX and subjected to streptavidin-based enrichment. After several washing steps, the modified peptides were selectively eluted from beads under 365 nm wavelength of UV light and subjected to LC-MS/MS-based proteomic analysis. Quantitation results were obtained from three biological replicates with two LC-MS/MS runs for each.

Table S6. Proteome-wide mapping LGs-targeting lysines in RAW264.7 cells. RAW264.7 cells were pretreated with or without 2µM LGs for 2h, and then incubated with 200 µM ONAyne for additional 2h. The ONAyne-labeled proteome was harvested and then processed into tryptic peptides. The resulting probe-labeled peptides were conjugated with both light (LGs treatment) and heavy (control) azido-UV-cleavable-biotin (Az-UV-biotin) reagents (1:1) via CuAAC reaction. The light and heavy 'Click' reaction mixtures were mixed equally in amount, and cleaned with SCX. The biotinylated peptides were captured with streptavidin and photoreleased for LC-MS/MS-based identification and quantification. High $R_{C/T}$ (=$R_{H/L}$) values are indicative of lysines that are less available after LGs treatment, suggesting potential target sites. Quantitation results were obtained from five biological replicates with one or two LC-MS/MS runs.

Methods

Reagents and antibodies

ONAyne was either synthesized as described previously[1] or purchased from a commercial source (https://www.nayuansu.com/pro/3082.html), ^{1}H NMR (400 MHz, $CDCl_3$) δ 9.81 (s, 1H), 2.80-2.73 (m, 4H), 2.64 (t, J = 7.3 Hz, 2H), 2.25 (td, J = 6.9, 2.6 Hz, 2H), 1.98 (t, J = 2.6 Hz, 1H), 1.82 (p, J = 7.1 Hz, 2H). ^{13}C NMR (400 MHz, $CDCl_3$) δ 207.95, 200.35, 83.47, 69.11, 41.04, 37.47, 34.77, 22.27, 17.73; Prostaglandin H_2 was purchased from Cayman Chemical (cat. no. 17020). STP alkyne was purchased from Lumiprobe Corporation (Cat. #: 30720). LGs were prepared as described previously via non-enzymatic rearrangement of prostaglandin H_2 in the presence of DMSO[2]; Iodoacetamide (cat. no. V900335), Tris[(1-benzyl-1H-1,2,3-triazol-4-yl)methyl]amine (TBTA) (cat. no. 678937), and sodium ascorbate (cat. no. A7631) were purchased from Sigma-Aldrich; Dithiothreitol (cat. no. A100281) was purchased from BBI Life Sciences; Light Azido-UV-Biotin (cat. no. EVU102), and Heavy Azido-UV-Biotin (cat. no. EVU151) were purchased from KeraFast; $CuSO_4$ (cat. no. C493-500) was purchased from Thermo Fisher Scientific; Sequencing grade trypsin (cat. no. V5113) was purchased from Promega. Rabbit monoclonal anti-Histone H2BK5ac (acetyl K5, cat. no. ab40886, 1:2000 for WB and 1:500 for IF), rabbit monoclonal anti-Histone H2BKac20 (acetyl K20, cat. no. ab177430, diluted at 1:1000 for WB and 1:250 for IF), rabbit monoclonal anti-Histone H2B (cat. no. ab1790, diluted at 1:500) were purchased from Abcam; Mouse monoclonal anti-β-Actin (cat. no. TA-09, diluted at 1:1000), goat anti-mouse IgG (cat. no. ZB-2306, diluted at 1:2500), goat anti-rabbit IgG (cat. no. ZB-2305, 1:2500), anti-rabbit IgG/AlexaFluor®594 (cat. no. ZF-0516, diluted at 1:250) and DAPI (cat. no. ZLI-9557, diluted at 1:1000) were purchased from ZSGB-Bio. Streptavidin beads (cat. no. 17-5113-01) were purchased from GE. MDH2 activity assay kit was purchased from Solarbio® Life Sciences (cat. no. BC1055)

Cell culture and treatment

RAW264.7 and MDA-MB-231 cells were purchased from the ATCC, cultured in DMEM supplemented with 10% FBS (Invitrogen), 1% penicillin-streptomycin (Invitrogen) and 1% Glutagro (Corning), maintained at 37°C in a 5% CO_2 humidified atmosphere. For LGs treatment, cells were grown to ~80% confluency and subjected to serum-deprivation overnight. Cells were then washed with fresh and pre-warmed serum-free medium and incubated with medium containing varying concentrations of LGs at 37°C for 2h. Since LGs represent a mixture of levuglandin (LG) D_2 and E_2, its concentration of treatment was estimated based on the adding concentration of prostaglandin H_2 in reaction solution.

Cytotoxicity assay

Cytotoxicity was evaluated with the CCK-8 assay kit (MCE, cat. no. HY-K0301) according to the manufacturer's protocol. RAW264.7 cells were treated with ONAyne or vehicle (DMSO) at indicated

concentrations for 2 h, 10 μL CCK-8 was added and the plates were incubated at 37 °C for 1 h. The plate was vortexed for 1 min and the absorbance at 540 nm was measured using a microplate reader.

ONAyne labeling

For *in situ* labeling, intact RAW264.7 cells with or without stimuli were incubated with 200 μM of ONAyne at 37°C for 2 h, harvested, and lysed in pre-chilled NETN buffer (50 mM HEPES (pH 7.6), 150 mM NaCl, and 1% IGEPAL) supplemented with 1 x protease and phosphatase inhibitors (Thermo Scientific, A32961). For *in vitro* labeling, RAW264.7 cells were lysed as above, filter with Thermo Scientific™ Zeba™ spin columns according to the manufactural instruction, and incubated with 200 μM of ONAyne at RT for 1 h; MDA-MB-231 cells were also lysed as above and were incubated with ONAyne as indicated concentrations (0.01 mM, 0.1mM, and 1mM) at 37°C for 1 h.

Preparing the ONAyne-labeled protein samples

The probe-labeled protein samples were processed using a chemoproteomic protocol previously reported [3]. Specifically, cell lysates incubated with 40 mM iodoacetamide at 37 °C for 1 h with light protection. Protein concentrations of lysate samples were then determined with the BCA assay (Pierce Thermo Fisher). For a typical ONAyne-based chemoproteomic analysis, 2 mg protein was used. To remove all the excess small molecules, proteins were then precipitated with a methanol-chloroform system (aqueous phase/methanol/chloroform, 4:4:1 (v/v/v)). In brief, proteins were collected at the phase interface as a solid white disk after centrifugation at 1,400g for 20 min at 4 °C. Liquid layers were discarded, and the protein was washed twice in prechilled methanol/chloroform (1:1, v/v), followed by centrifugation at 16,000g for 10 min at 4 °C. The precipitated proteins were resuspended with 50 mM ammonium bicarbonate containing 0.2 M urea with sonication (10 sec with 20% output, repeat three times) and digested with trypsin at a 1:50 (enzyme/substrate) ratio overnight at 37°C. The tryptic digests were desalted with HLB extraction cartridges (Waters) as described previously and evaporated to dryness under vacuum. The dried peptides were resuspended in a water solution containing 30% acetonitrile (MeCN). CuAAC reaction was then performed by subsequently adding 1 mM either light or heavy Azido-UV-biotin (1 μL of a 40 mM stock), 10 mM sodium ascorbate (4 μL of a 100 mM stock), 1 mM TBTA (1 μL of a 50 mM stock, and 10 mM $CuSO_4$ (4 μL of a 100 mM stock). After 2h incubation at RT, the light and heavy isotopic tagged samples were then mixed immediately following CuAAC reaction and cleaned with strong cation exchange (SCX). In brief, the sample was diluted into SCX loading buffer (5 mM KH_2PO_4, 25% MeCN, pH 3.0), passed through the SCX spin columns (The Nest Group) and washed with several column volumes of loading buffer. The retained peptides were eluted with a series of high-salt buffers containing 400 mM NaCl. The eluted peptides were diluted 10x with 50 mM sodium acetate buffer (NaAc, pH 4.5) and then subject to the enrichment with streptavidin agarose beads (GE). After 2 h of incubation at RT, streptavidin beads were washed

with 50 mM NaAc (pH4.5), 50 mM NaAc containing 2 M NaCl (pH4.5), and deionized water twice each with vortexing and/or rotation to remove non-specific binding substances, then resuspended in 25 mM ammonium bicarbonate, transferred to glass tubes (VWR), and irradiated with 365 nm UV light (Entela, Upland, CA) for 2 h at RT with magnetic stirring. The supernatant was collected, dried under vacuum, and stored at −20°C until LC-MS/MS analysis.

Liquid chromatography-tandem mass spectrometry (LC-MS/MS)

LC-MS/MS analyses were performed on a Q Exactive^{TM} HF-X (Thermo Fisher Scientific) equipped with a UltiMateTM 3000 RSLCnano system (Thermo Fisher Scientific) for online sample handling and peptide separations. Then 6 µL of sample was loaded onto a fused-silica nano-ESI column (360 µm OD × 150 µm ID) with a needle tip (3~5 µM) packed to a length of 15 cm with a C18 reverse phase resin (AQ 1.9µm, 120Å, ReproSil-Pur). The peptides were separated using an 88min linear gradient from 6% to 95% buffer B (80% MeCN) equilibrated with buffer A (0.1% formic acid) at a flow rate of 600 nL/min across the column. The scan sequence for the Orbitrap began with an MS1 spectrum (Orbitrap analysis, resolution 120,000, scan range of 350-1550 *m/z*, AGC target 3×10^6, maximum injection time 20ms, dynamic exclusion of 15 seconds). The "Top25" precursors was selected for MS2 analysis, in which precursors were fragmented by HCD prior to Orbitrap analysis ((N)CE 27, AGC target 2×10^4, maximum injection time 30ms, resolution 15,000, and isolation window: 1.6 Da). The MS data sets have been deposited to the ProteomeXchange Consortium via the PRIDE partner repository with the dataset identifier PXD028270 (Username: reviewer_pxd028270@ebi.ac.uk; Password: ML348wpA).

pChem analysis

The MS data was directly imported into pChem (http://pfind.ict.ac.cn/software/pChem/index.html) that could automatically invoke an efficient open-search engine[4] to generate all possible mass shifts within the given range (±1, 000 Da by default) at the PSM (peptide spectrum match)-level. Common modifications (i.e., oxidation of methionines and carbamidomethylation of cysteines) were predefined to improve search sensitivity. Then, all mass shifts were calibrated with the calculated system error and unified to generate modification candidates. The latter were automatically paired according to the isotopic mass difference between a pair of light and heavy PDMs ($^{12}C_6$/$^{13}C_6$, 6.020132Da). Furthermore, all non-PDMs and unrealistic modifications were filtered out by 1) neglecting a measured mass difference out of the range of [6.020132 - 0.001, 6.020132 + 0.001] Da, corresponding to 166 ppm mass tolerance for the theoretical value; 2) discarding less abundant modification candidates with the PSM counting number lower than 5% of total; 3) removing the modifications with masses lower than 200 Da. The retained high-confident PDMs were then exported into a summary file containing

many key characteristics for each (incl. accurate masses, site-specific localization probability, **Table S1**) and a heat map showing the amino acid localization distribution for each PDM (**Fig. S9**).

Targeted identification and quantification

For targeted database search and quantification, the MS data was imported into pFind 3.0 and processed as previously described[4]. Specifically, raw data files were searched against *Mus musculus* (for RAW264.7) or *Homo sapiens* (for MDA-MB-231) Uniprot canonical database using pFind studio (http://pfind.ict.ac.cn/software/pFind/index.html)[4]. Precursor ion mass and fragmentation tolerance were set as 10 ppm and 20 ppm, respectively. The maximum number of modifications and missed cleavages allowed per peptide were both set as three. For all analyses, mass shifts of common modifications (i.e., methionine oxidation + 15.9949 Da; and cysteine carbamidomethylation + 57.0214 Da) and ONAyne-derived modifications listed in **Figure S6** were searched as variable modifications. An isotopic mass difference of 6.020132 Da ($^{12}C_6$/$^{13}C_6$) on probe-derived modifications was used for quantification. The FDRs were estimated by the program from the number and quality of spectral matches to the decoy database. The FDRs at spectrum, peptide, and protein level were < 1%. Quantitative analyses were performed using pQuant[5] , which calculates heavy to light ratios based on each identified MS scan with a 15 ppm-level m/z tolerance window and assigns an interference score (Int. Score) to each value from zero to one. The median values of probe-modified peptide ratios with σ less than or equal 0.5 were considered to calculate site-level ratios. For peptides exhibiting a $\geqslant$ 90% reduction in MS1 peak intensity, a maximal ratio of 10 was assigned. Overall, quantification results were obtained from at least three biological replicates.

Motif analysis

The consensus sequence motifs of hyper-reactive lysine residues determined by either ONAyne-based or STP alkyne-based ABPP were visualized by pLogo[6], a linear sequence prediction algorithm based on their statistical significance ($P \leq 0.05$).

MDH2 activity assay

RAW264.7 cells pre-treated with or without LGs (2 μM) were washed with cold PBS twice, harvested by centrifugation, and subjected to MDH2 activity assays according to the manufacturer's protocol. In brief, ~1×10^6 cells were resuspended in 200 μL pre-chilled lysis buffer. The supernatants were collected by centrifugation at 8000 g for 10 min at 4°C, incubated subsequently with reagents A-C, and measured immediately at 340 nm wavelength using the pre-calibrated UV spectrophotometer.

Immunoprecipitation

RAW264.7 cells treated with or without LGs were labeled with ONAyne *in situ*, washed twice with ice cold 1X PBS, harvested, and then lysed. Cell lysates (1 mg/mL, 78 μL) were incubated with the addition of 2 μL click reaction mixture (40 mM N_3-Biotin stock solution/100 mM sodium ascorbate/50 mM TBTA /100 mM $CuSO_4$, 5:10:2:10, v/v/v/v). After 2h incubation at RT, the mixture was incubated with 40 μL streptavidin beads at 4 °C overnight, and then washed with 1 mL PBS twice. The resulting beads were resuspended with 40 μL loading buffer (1×, Beyotime, China) and boiled for 5 min. The supernatant was collected by centrifugation and further incubated with 10 μL loading buffer (5×) prior to SDS-PAGE and western blotting.

Western blotting

Equal amounts of protein samples (20 μg) were mixed with loading buffer, boiled for 5 min, and then resolved by SDS-PAGE. Proteins were transferred to NC membranes (0.22 μm, Millipore, U.S.), blocked with 5% milk in TBST (20 mM Tris-HCl, pH 7.6, 150 mM NaCl, and 0.1% v/v Tween-20) at RT for 1h and probed with the indicated primary antibodies overnight at 4°C. After incubation, the membranes were washed three times with TBST containing 5% milk and then incubated with the appropriate HRP-conjugated specific secondary antibodies. Blots were visualized on a Tanon 5200 scanner (Shanghai, China), and analyzed using GelCap software using ECL chemiluminescence (CWBIO, cat. no. CW0049S).

Immunofluorescence imaging

RAW264.7 cells were cultured onto confocal dishes at a density of 1×10^5 cells/mL and treated with or without LGs. After 2h incubation at 37°C, cells were washed with pre-chilled PBS (x3), fixed in 4% paraformaldehyde at RT for 30 min, washed again with pre-chilled PBS (x3), and permeabilized with 0.2% TritonX-100 at 4°C for 10 min. Then cells were blocked in 3% BSA for 1h at RT. Fixed cells were then incubated with the antibodies as indicated, followed by exposure to the appropriate fluorescent secondary antibodies. Finally, stain nucleus with DAPI (ZSGB, ZLI-9557, diluted at 1:1000). Fluorescence was observed with a laser-scanning microscope (ZEISS, LSM880 ELYRAS.1).

Synthesis of ONAyne

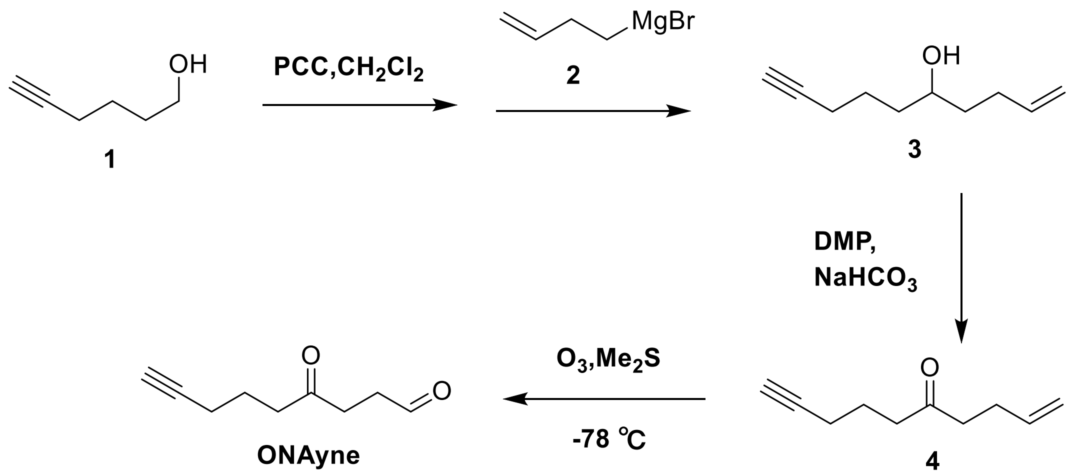

Scheme S1 Synthetic route for ONAyne (**5**).

General information

All commercially available chemicals were obtained from Aldrich, Energy Chemical, Aladdin, Macklin and were used without further purification, except where noted. All NMR spectra were recorded on a 400 MHz Bruker NMR spectrometer in $CDCl_3$ with TMS as internal standard for protons and solvent signals as internal standard for carbon spectra. Chemical shift values are mentioned in δ(ppm) and coupling constants (J) are given in Hz.

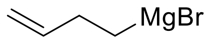

Preparation of Grignard reagent (2).

Magnesium (0.58g, 23.8mmol), iodine (3.0g, 23.6mmol) and solution of 4-bromo-1-butene (1.63g, 12.1mmol) in anhydrous THF (2mL) were added to anhydrous THF (50 mL) under argon. The suspension was heated slightly to initiate the reaction, then refluxed for 2 h, and cooled to room temperature to obtain **2**.

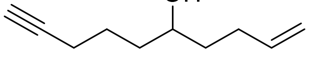

Synthesis of dec-1-en-9-yn-5-ol (3).

The hex-5-yn-1-ol (**1**) (1.0 g, 10.2 mmol) was dissolved in CH_2Cl_2 (50 mL) and in cold bath. The solution was stirred and pyridinium chlorochromate (PCC) (3.3 g, 15.2 mmol) was added. The mixture was stirred at room temperature until the material was consumed, then the mixture was filtered and concentrated. Obtain crude aldehyde and dissolve in THF (50mL) at -78°C and stirred, **2** was slowly

added with an airtight syringe. After stirring at -78°C for 30 min, the reaction mixture was quenched with saturated NH_4Cl aqueous solution and extracted with EtOAc. The combined organic phase was dried (Na_2SO_4) and evaporated. Flash chromatography of the residue over silica gel, using PE/EtOAc (3:1), gave **3** (oily, 0.84 g, 5.5 mmol, yield 54%).

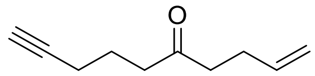

***Synthesis of dec-1-en-9-yn-5-one (4)*.**

To a stirred solution of **3** (0.8 g, 5.3 mmol) in CH_2Cl_2 (30 mL) at 0° C, $NaHCO_3$ (1.34 g, 16 mmol) and Dess-Martin Periodinane (DMP) (8.2 mmol) were sequentially added. After stirring at RT for 30 min, the reaction was quenched with saturated aqueous $Na_2S_2O_3$ at 0°C. The aqueous phase was extracted with CH_2Cl_2 (5×30 mL). The combined organic phase was dried (Na_2SO_4) and evaporated. Flash chromatography of the residue over silica gel, using PE/EtOAc (2:1), gave **4** (oily, 0.71 g, 4.73 mmol, yield 89%).

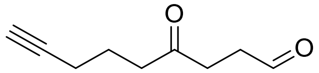

***Synthesis of 4-oxonon-8-ynal (ONAyne)*.**

Ozone was bubbled to the solution of **4** (0.65g, 4.33mmol) in CH_2Cl_2 (30 mL) for 0.5 h. Then Me_2S (2.7g, 43 mmol) was added to the reaction solution, the mixture was heated to RT and stirred for 6 h. The mixture evaporated and purified by flash chromatography of the residue over silica gel, using 5:1 PE/EtOA, gave ONAyne (oily, 0.47 g, 3.1 mmol, 70% yield).

NMR spectra

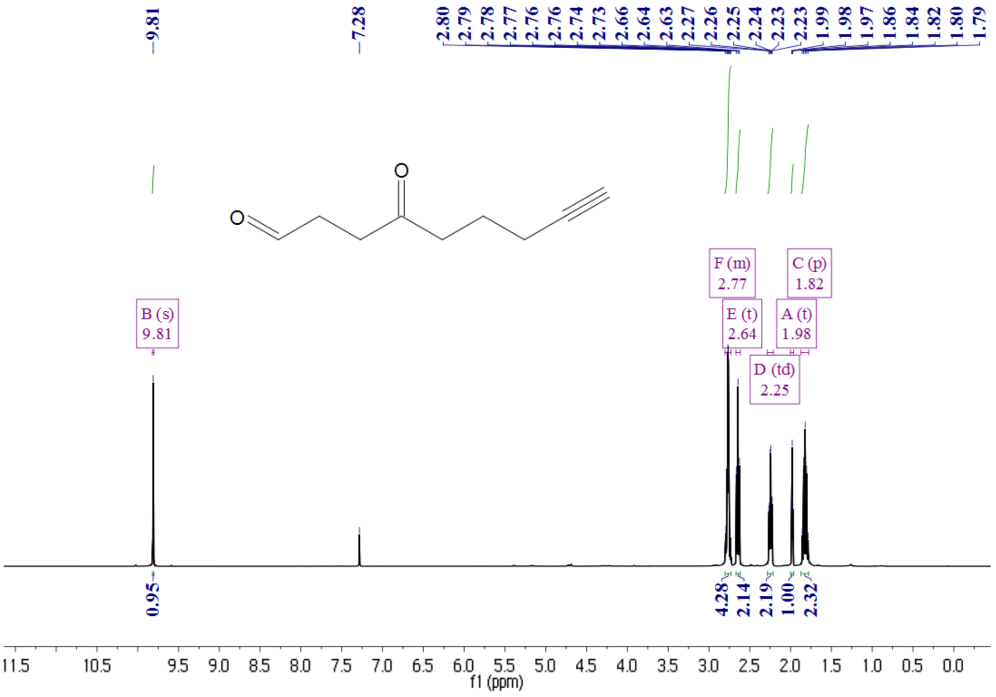

^{1}H NMR spectra of ONAyne.

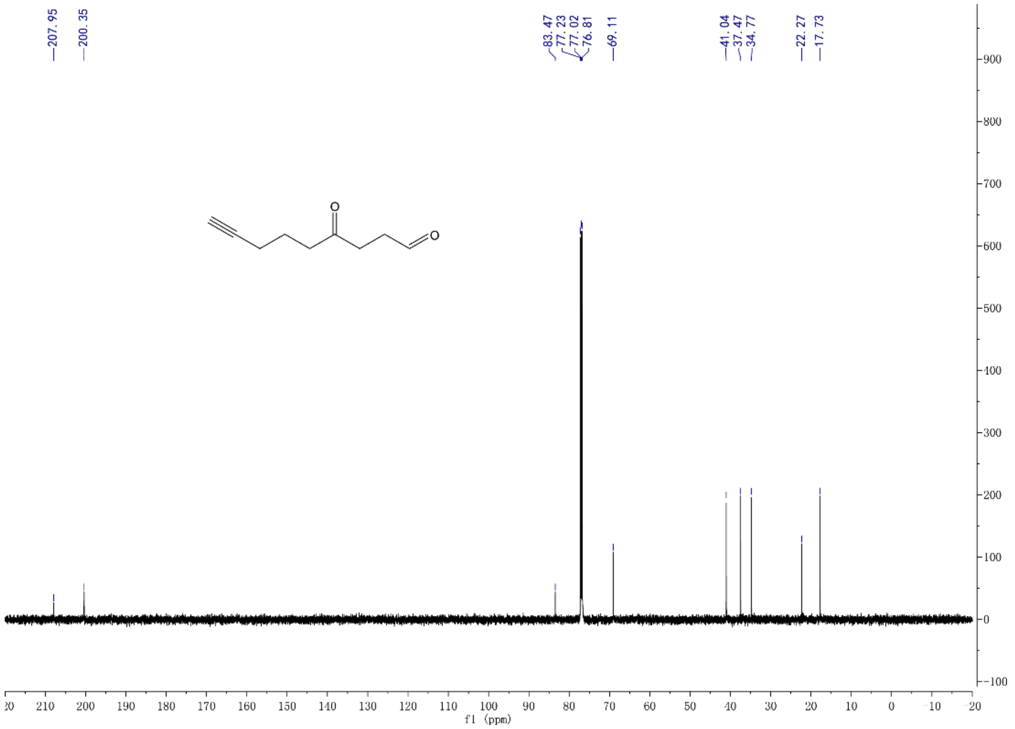

^{13}C NMR spectrum of ONAyne.

EI-MS spectrum

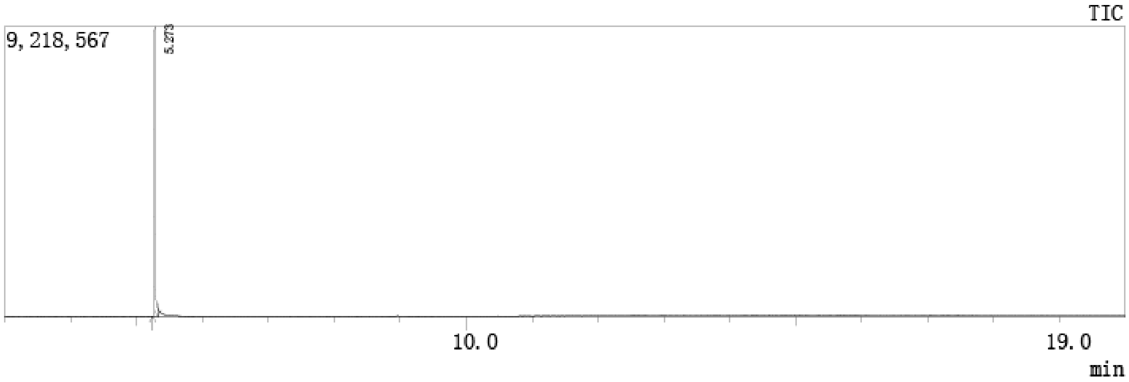

Peak Report TIC

Peak#	R.Time	I.Time	F.Time	Area	Area%	Height	Height%
1	5.273	5.245	5.325	10545790	100.00	9197178	100.00
				10545790	100.00	9197178	100.00

Spectrum

Peak#:1 R.Time:5.273(Scan#:455)
MassPeaks:312
RawMode:Averaged 5.265-5.275(454-456)
BG Mode:Calc. from Peak Group 1 - Event 1

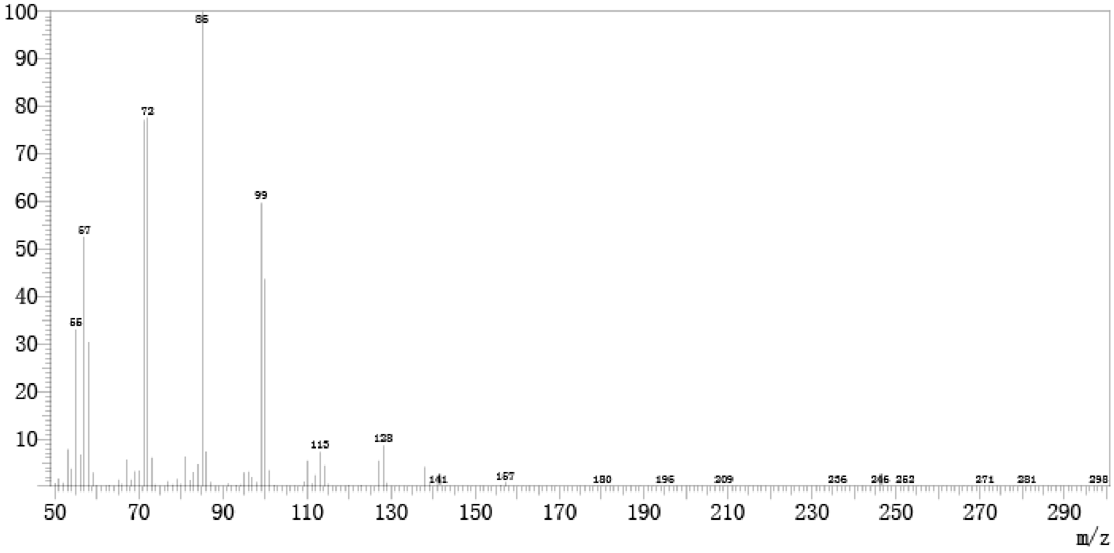

Gas chromatography tandem mass spectrometry (GC-MS) analysis of ONAyne.

References

[1] W. Bao, Y. Tao, J. Cheng, J. Huang, J. Cao, M. Zhang, W. Ye, B. Wang, Y. Li, L. Zhu, C. S. Lee, *Org. Lett.* **2018**, *20*, 7912–7915.

[2] S. S. Davies, V. Amarnath, C. J. Brame, O. Boutaud, L. J. Roberts, *Nat. Protoc.* **2007**, *2*, 2079–2091.

[3] L. Fu, Z. Li, K. Liu, C. Tian, J. He, J. He, F. He, P. Xu, J. Yang, *Nat. Protoc.* **2020**, *15*, 2891–2919.

[4] H. Chi, K. He, B. Yang, Z. Chen, R. X. Sun, S. B. Fan, K. Zhang, C. Liu, Z. F. Yuan, Q. H. Wang, S. Q. Liu, M. Q. Dong, S. M. He, *J. Proteomics* **2015**, *125*, 89–97.

[5] C. Liu, C. Q. Song, Z. F. Yuan, Y. Fu, H. Chi, L. H. Wang, S. B. Fan, K. Zhang, W. F. Zeng, S. M. He, M. Q. Dong, R. X. Sun, *Anal. Chem.* **2014**, *86*, 5286–5294.

[6] J. P. O'Shea, M. F. Chou, S. A. Quader, J. K. Ryan, G. M. Church, D. Schwartz, *Nat. Methods* **2013**, *10*, 1211–1212.
